# High sensitivity organic inorganic hybrid X-ray detectors with direct transduction and broadband response

**DOI:** 10.1038/s41467-018-05301-6

**Published:** 2018-07-26

**Authors:** H. M. Thirimanne, K. D. G. I. Jayawardena, A. J. Parnell, R. M. I. Bandara, A. Karalasingam, S. Pani, J. E. Huerdler, D. G. Lidzey, S. F. Tedde, A. Nisbet, C. A. Mills, S. R. P. Silva

**Affiliations:** 1 Department of Electrical and Electronic Engineering, Advanced Technology Institute, University of Surrey, Guildford, Surrey, GU2 7XH UK; 2 Department of Physics and Astronomy, University of Sheffield, Hicks Building, Sheffield, S3 7RH UK; 3 Department of Physics, University of Surrey, Guildford, Surrey, GU2 7XH UK; 4 Siemens Healthineers GmbH, Technology Centre, 91058 Erlangen, Germany; 5 Department of Medical Physics, Royal Surrey County Hospital NHS Foundation Trust, Egerton Road, Guildford, GU2 7XX UK

## Abstract

X-ray detectors are critical to healthcare diagnostics, cancer therapy and homeland security, with many potential uses limited by system cost and/or detector dimensions. Current X-ray detector sensitivities are limited by the bulk X-ray attenuation of the materials and consequently necessitate thick crystals (~1 mm–1 cm), resulting in rigid structures, high operational voltages and high cost. Here we present a disruptive, flexible, low cost, broadband, and high sensitivity direct X-ray transduction technology produced by embedding high atomic number bismuth oxide nanoparticles in an organic bulk heterojunction. These hybrid detectors demonstrate sensitivities of 1712 µC mGy^−1^ cm^−3^ for “soft” X-rays and ~30 and 58 µC mGy^−1^ cm^−3^ under 6 and 15 MV “hard” X-rays generated from a medical linear accelerator; strongly competing with the current solid state detectors, all achieved at low bias voltages (−10 V) and low power, enabling detector operation powered by coin cell batteries.

## Abstract (web summary)

X-ray detectors based on low-cost organic semiconductors have inherently low detector sensitivity due to poor X-ray to charge conversion and charge collection. Here, the authors demonstrate a flexible, high-sensitivity X-ray detector based on an organic bulk heterojunction-nanoparticle composite.

## Introduction

X-rays are widely used in homeland security, therapeutic and diagnostic healthcare and industrial process control (e.g. pharmaceuticals) with each application necessitating specific detector requirements^1^. For example, direct conversion detectors based on materials such as amorphous selenium^2^ are currently used in mammography, but are limited by their low X-ray attenuation for energies higher than 50 keV. Detectors based on p-type silicon with its high radiation-damage resistance are used in radiotherapy for dose measurement or beam imaging^3^. However, their propensity to damage from accumulated dose and drift due to environmental effects makes these less useful for beam calibration^4^. High-quality single crystal Cd(Zn)Te^5^ is used for homeland security screening, but suffers from being limited to small dimensions, high cost, charge carrier trapping and high-voltage operation (>500 V). Similarly, X-ray detection in the non-destructive evaluation sector is currently dominated by CsI (Tl) scintillator screens coupled to a-Si which, despite their high stopping power and spatial resolution, are limited to sizes less than 60 × 60 cm^2^. Therefore, there is a demand for broadband, high sensitivity, low-cost radiation detectors, which current inorganic detectors fail to fulfil.

Organic semiconductors can be fabricated over large areas in a flexible format, enabling conformability to complex structures at low cost and are now commercialized for photovoltaics, displays etc^6^. There is increasing attention given to organic photodetectors for X-ray detection^7,8^. This often involves the coupling of scintillator screens with organic photodiodes^9^, insertion of high-atomic number (*Z*) nanoparticles (NPs)^10^, quantum dots^11^ or scintillator particles into organic diodes^12^, or the use of thin film organic semiconductors^8^ or crystals^13^. Of these, the use of X-ray scintillators is often preferred as this enables the already mature organic photodetector technologies to be adapted for X-ray detection. However, the absorption of light by the organic semiconductor forms bound electron–hole pairs (excitons), which need to be dissociated^12^ resulting in significant losses, limiting detector sensitivity as opposed to a direct conversion process.

Here, we introduce a broadband, direct, X-ray detector concept based on a thin film, hybrid semiconductor diode consisting of an organic bulk heterojunction (BHJ)—bismuth oxide (Bi_2_O_3_) NP composite. These direct X-ray detectors demonstrate high sensitivities of 1712 µC mGy^−1^ cm^−3^ under 50 kV soft X-rays and ~30 and 58 µC mGy^−1^ cm^−3^ under 6 and 15 MV hard X-rays. Furthermore, we also demonstrate a flexible detector based on the same device concept which offer a high sensitivity of 280 µC  mGy^−1^ cm^−3^. More importantly, these sensitivities are achieved at −10 V.

## Results

### X-ray response of BHJ-NP detectors

**Fig. 1:**
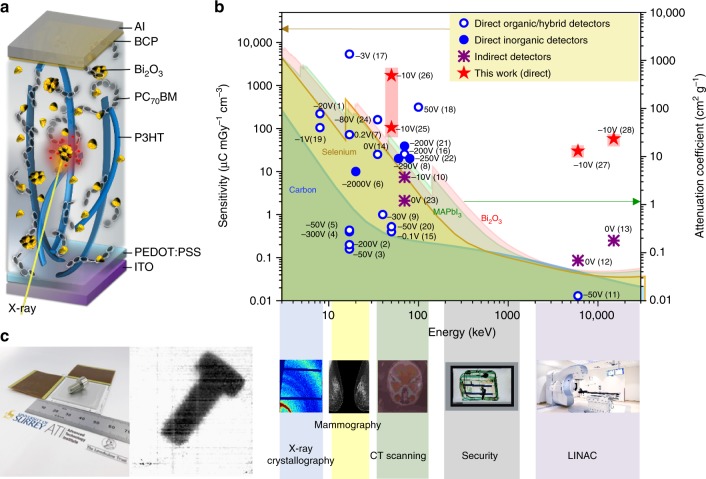
X-ray detector overview. **a** Device schematic structure. **b** Performance comparison of current solid state X-ray detectors—(1)^13^, (2)^10^, (3)^36^, (4)^37^, (5)^38^, (7)^8^, (9)^11^, (11)^3^, (14)^39^, (15)^40^, (16)^7^, (17)^41^, (18)^42^, (19)^43^, (20)^44^, and (24)^20^ are direct detectors, (6)^45^, (8)^46^, (21)^7,47^ and (22)^48^ are inorganic detectors, and (10)^12^, (12)^9^, (13)^25^ and (23)^49^ are indirect detectors—with the technology developed in this work—(25) Bi_2_O_3_-40, (26) Bi_2_O_3_-80, (27) Bi_2_O_3_-40 and (28) Bi_2_O_3_-40. The operating voltage is given adjacent to each data point. The total attenuation coefficient values of carbon, selenium, methylammonium lead iodide (MAPbI_3_) and Bi_2_O_3_ are given as shaded areas showing the previous limits to detector technology based only on bulk attenuation processes. **c** An X-ray imager based on the hybrid X-ray detector and 70 kV X-ray image of a bolt taken using the X-ray imager

The device compromises of a diode architecture where the BHJ-NP composite is sandwiched between indium tin oxide (ITO) and aluminium (Al) electrodes (Fig. 1a). Here, the introduction of the Bi_2_O_3_ (*Z* = 83 for Bi) is utilised to increase the X-ray attenuation^14^. We have chosen Bi_2_O_3_ from the many metal oxides available based on its direct conversion of X-rays and lower environmental impact and health risks when compared to, for example, high *Z* Pb-based semiconductors. Given its existing use as a non-toxic dental material such as in the case of hydraulic silicate cements^15^ with an opacity to X-rays makes it an ideal candidate for our application. Regioregular poly(3-hexylthiophene-2,5-diyl) (P3HT) and [6,6]-Phenyl C_71_ butyric acid methyl ester (PC_70_BM) were selected as the BHJ system. The formation of nanoscale diodes throughout the volume of the BHJ, in close proximity to the NPs leads to an in-built depletion region, with local electric fields as high as ~200 V µm^−1^^16^, which has been experimentally quantified with Fourier-transform IR-absorption spectroscopy for the P3HT:PCBM system. This is further enhanced by dielectric inhomogeneities in the material^17,18^. The above factors, in combination with the high crystallinity of P3HT:PC_70_BM enables efficient electron and hole extraction from the entirety of the depleted active layer under low reverse-bias voltages (<10 V). The above factors enable an X-ray detector with high sensitivity that strongly competes with all existing solid state X-ray detector technologies, under low voltages as well as over a broad X-ray energy range (Fig. 1b and Supplementary Figure 1) with potential applications in X-ray imaging as shown from a prototype imager developed through this work (Fig. 1c).

For optimisation of the detector, the NP loading within the device active volume was increased in order to increase the X-ray attenuation by varying the Bi_2_O_3_ content in the parent solution (0, 10, 20, 30, 40, 50, 60, 70 and 80 mg ml^−1^: X mg ml^−1^ is noted as Bi_2_O_3_-X). It is worth to note that the highest NP-loaded device which could be fabricated using the given procedure is Bi_2_O_3_-80, due to the formation of cracks during the annealing process at higher loadings beyond Bi_2_O_3_-80. However, with the appropriate selection of the organic bulk heterojunction and with tuning of the solvent used, much higher Bi_2_O_3_-loaded device fabrication maybe possible. Assuming a periodic structure for the NPs within the BHJ, this enables the unit cell dimensions to be reduced from 72 nm for Bi_2_O_3_-40 to 64 nm for Bi_2_O_3_-80 (Supplementary Figure 2 and Supplementary Note 1). The detectors with thicknesses of ~10–30 μm (Supplementary Figure 3) demonstrate dark current densities in the range of 10^−4^ (Bi_2_O_3_-80) to 10^−6^ (Bi_2_O_3_-0) A cm^−2^ at −10 V, and ~1 and ~40 nA cm^−2^ under 0 and −1 V, respectively (Supplementary Figure 4a). We note recent work in the literature where the dark current can be tuned to meet industrial requirements through appropriate BHJ selection as a promising route for further improvements ^19^.

**Fig. 2:**
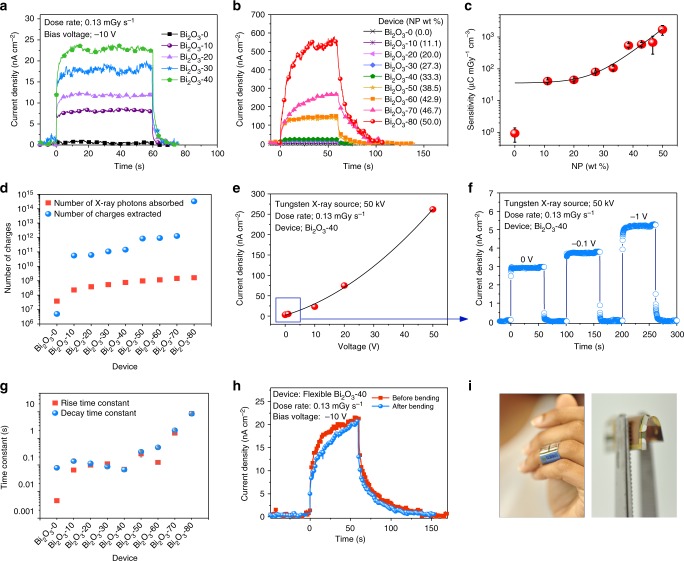
Detector performance under X-ray irradiation. **a** X-ray photocurrent densities for devices with different Bi_2_O_3_ loadings from Bi_2_O_3_-0 to Bi_2_O_3_-40 and **b** from Bi_2_O_3_-0 to Bi_2_O_3_-80. **c** Averaged sensitivity values for six devices and the error bars represent the range of the sensitivity values. **d** Comparison between number of X-ray photons absorbed by each device and the number of charges extracted. **e** The voltage dependence of the Bi_2_O_3_-40 device, **f** X-ray photocurrent response of the Bi_2_O_3_-40 detector under 0, −0.1 and −1 V biases. **g** Rise and decay time constants (the error bars represent the standard error shown with respect to the fitted curves) for detectors with increasing Bi_2_O_3_ loadings under −10 V bias. **h** X-ray photocurrent response before and after bending for a flexible Bi_2_O_3_-40 device. **i** A prototype X-ray detector integrated into a plaster and detector bend radius (0.3 cm)

Visible light photocurrent measurements are a useful tool in determining whether NP incorporation disrupts the BHJ phase separation thereby impeding charge transport. The lack of significant variation in the visible light photocurrent response for different Bi_2_O_3_ loadings indicates that the phase separation within the BHJ remains undisturbed (Supplementary Figure 4b). The X-ray photocurrent response of the detectors tested under a 50 kV X-ray source at −10 V bias, demonstrates a linear increase with increasing NP loading from Bi_2_O_3_-0 to Bi_2_O_3_-40 (Fig. 2a, b), followed by a non-linear increase for Bi_2_O_3_-60 and upwards. The X-ray sensitivity (*S*) depends on the amount of X-rays stopped, which depends on both the device cross section and its thickness, and hence, the sensitivity of the detector is calculated by:

1 $$S = \frac{{{\int} {\left[ {I_{{\mathrm{X{\operatorname{-}}ray}}}\left( t \right) - I_{{\mathrm{dark}}}} \right]{\mathrm{d}}t} }}{{D \times V}}$$

where, *I*_X-ray_ and *I*_dark_ are the current under X-ray irradiation and in the dark respectively, *D* is the dose and *V*, the detector volume. For the detectors studied herein, *S* increases from 41 (Bi_2_O_3_-10) to 1712 (Bi_2_O_3_-80) µC mGy^−1^ cm^−3^ (Fig. 2c). We note that Ciavatti et al.^20^ recently reported Bi_2_O_3_ NP-loaded PFO polymer-based diodes for X-ray detection where the highest *S* observed was 160 µC mGy^−1^ cm^−3^ which is slightly higher than the *S* of Bi_2_O_3_-40 (105 µC mGy^−1^ cm^−3^). Despite the similarity in magnitude of these *S* values, we note that the former work employed a 35 kV Mo target as opposed to the 50 kV W target used in this work. As stated and shown schematically (Fig. 1b), the X-ray attenuation significantly improves by approximately one order of magnitude as the X-ray spectrum shifts to lower energies which is expected to result in the nearly similar *S* values for the Bi_2_O_3_ NP-loaded PFO polymer-based diodes and the Bi_2_O_3_-40 diodes. We expect the *S* values for the Bi_2_O_3_-40 diodes to be higher if measured under low kV sources due to the enhanced X-ray attenuation as a result of increase in the mass attenuation, especially under high NP loading as well as due to the use of the BHJ thick films which enables a more balanced carrier transport. The combination of the direct conversion of X-ray photons to charge carriers as well as the very high electric fields at the hybrid interfaces adjacent to the NPs, is a necessity for the much improved currents observed. The number of charges extracted (as calculated from the X-ray photocurrent response) vs. the number of X-ray photons absorbed (calculated based on the bulk X-ray attenuation model—Supplementary Figure 5) indicates a ~× 10^3^ enhancement in charge collection efficiency (CCE) than expected from the bulk attenuation model for Bi_2_O_3_-10 to Bi_2_O_3_-70, and a ~× 10^5^ CCE increase for Bi_2_O_3_-80 (Fig. 2d). In comparison, the Bi_2_O_3_-0 system shows nearly an order of magnitude lower CCE.

The voltage dependence of the BHJ-NP X-ray detectors display a linear behaviour for low reverse-bias voltages (<10 V) with sensitivity of 0.13 µC mGy^−1^ cm^−3^ for low-voltage bias (−2.5 V) indicating its suitability for portable, real time radiation monitoring, powered by coin cell batteries or by indoor lighting. An X-ray photocurrent response was also obtained under short-circuit conditions (at 0 V) (Fig. 2e, f). A non-linear X-ray current response is observed for higher biases due to space charge-limited conduction with a three orders of magnitude improvement observed under −50 V. Further, as the voltage increases, the X-ray response increases while retaining the dark currents of 6 and 41 nA cm^−2^ (at −0.1 and −1 V, respectively). The sensitivity of 0.13 µC mGy^−1^ cm^−3^ under reverse-bias voltages of <3 V indicates the suitability of this technology for portable, real time radiation monitoring, with power provided by coin cell batteries or by indoor light harvesting photovoltaic cells.

Figure 2g shows that the rise time (to 90% of the maximum signal) and the fall time (to 10% of the maximum signal) increases as the Bi_2_O_3_ loading increases. The slow response under reverse bias is due to the trap states generally present in metal oxide surfaces^21^ and between the metal/semiconductor interfaces^22^. Further development in passivation of these defects is expected to significantly improve the response time of these detectors. We note that the rise and fall times are significantly reduced to less than 100 ms when the diodes are operated under 0 V. Another important detector metric is the linearity of the detector response, which enables the rapid determination of the X-ray dose, especially for dosimetry. Supplementary Figure 6 shows a representative X-ray photocurrent response curve for the Bi_2_O_3_-40 device in which an excellent linear response is observed.

An area of significant interest of X-ray imaging is the development of flexible, conformable imagers, a feature not enabled by current digital flat panel X-ray detectors. In order to evaluate the performance of the BHJ-NP detectors under deformation, we fabricated the optimized Bi_2_O_3_-40 X-ray detectors on a flexible substrate. The detectors demonstrate a nearly unchanged, high sensitivity of 280 µC mGy^−1^ cm^−3^ (Fig. 2h**)** both prior to, and after undergoing 10 bend cycles of ~3 mm bend radius (Fig. 2i). A slight variation in the rise and decay constant was observed possibly due to the poor mechanical properties of the contact materials or the use of a fullerene-based acceptor, as opposed to an all polymer-based BHJ, which might have deteriorated the mechanical properties of the BHJ^23^. Based on our concept of hybrid organic–inorganic materials for X-ray detectors, many new and more suitable combinations can now be examined for future detectors.

**Fig. 3:**
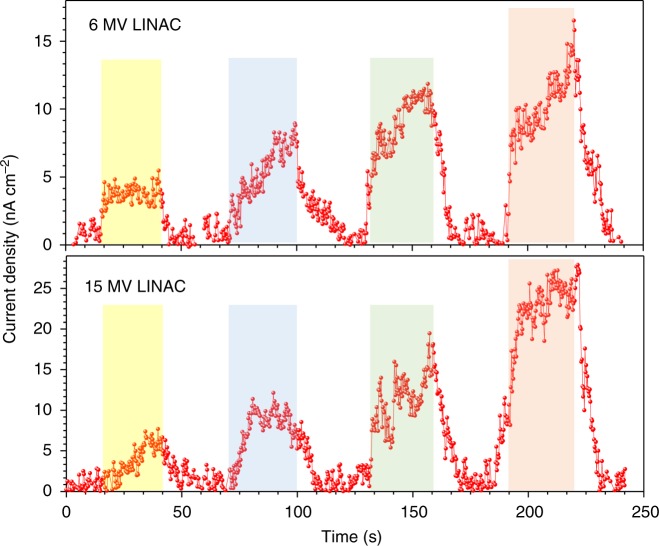
Hard X-ray irradiation. Response of a Bi_2_O_3_-40 device under linear accelerator (LINAC) generated 6 and 15 MV X-rays, the consecutive peaks represent the response to dose rates of 114, 227, 340 and 454 µGy s^−1^

A major potential application for such conformable X-ray detectors is, as dosimeters to be used in combination with a medical linear accelerator (LINAC), which are widely used for cancer therapy. The use of a conformable dosimeter as an in vivo detector on the surface of the patient or within a body cavity is highly likely to enable a more accurate X-ray delivery to patients thereby minimizing additional normal tissue damage as well as potential risks related to secondary cancer induction. A recent review in this field has recommended that all the radiotherapy treatments with curative intent should be verified through in vivo dose measurements in combination with pre-treatment checks^24^. As such, our optimized Bi_2_O_3_-40 detectors were tested under 6 and 15 MV X-rays from a medical LINAC (Fig. 3). Under a 114 µGy s^-1^ dose rate and −10 V reverse bias, the detector delivered a sensitivity of 30 and 58 µC mGy^−1^ cm^−3^ for 6 and 15 MV X-rays, respectively. These values are nearly ×100 higher than those reported for organic photodetectors tested under 6 and 15 MV LINAC X-rays using Gd_2_O_2_S:Tb as a scintillator^9,25^, therefore demonstrating that the BHJ-NP architecture developed here is a promising dosimeter concept for accurate dose delivery. It is noted that the Bi_2_O_3_-40 device did not show a noticeable performance degradation when exposed to 6 and 15 MV X-rays over several X-ray exposure cycles which results in a cumulative exposure dose of 0.15 Gy.

### Structural characterisation

**Fig. 4:**
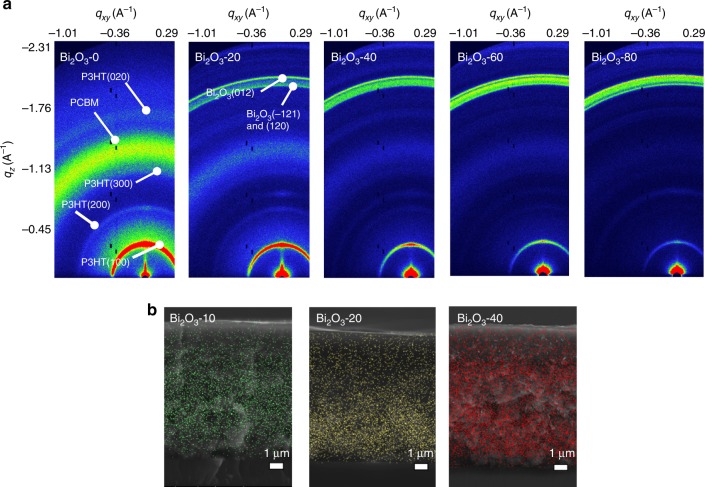
X-ray detector structural characterisation. **a** Grazing incidence wide angle X-ray scattering images of Bi_2_O_3_-0, Bi_2_O_3_-20, Bi_2_O_3_-40, Bi_2_O_3_-60 and Bi_2_O_3_-80. Here, the position of the poly(3-hexylthiophene-2,5-diyl) (P3HT), [6,6]-Phenyl C_71_ butyric acid methyl ester (PCBM) and bismuth oxide (Bi_2_O_3_) resultant peaks are indicated. **b** Scanning electron microscopy cross sections of Bi_2_O_3_-10, Bi_2_O_3_-20 and Bi_2_O_3_-40 overlaid with energy dispersive X-ray spectroscopy imaging of the Bi_2_O_3_ nanoparticles (false coloured for clarity)

We examined the impact of the Bi_2_O_3_ NP inclusion on the structural properties of the P3HT:PC_70_BM through Grazing Incidence Wide Angle X-Ray Scattering (GI-WAXS) (Fig. 4a). While the intensity of the Bi_2_O_3_ diffraction rings increases with increasing Bi_2_O_3_ loading, a high P3HT crystallinity is also observed for all films, peaking at Bi_2_O_3_-40, with a slight reduction in the Bi_2_O_3_-60 to Bi_2_O_3_-80 devices. Further structural characterisation is explained in Supplementary Figures 7, 8 and Supplementary Note 2, 3.

Energy dispersive X-ray analysis carried out on the BHJ-NP film cross sections (Fig. 4b) shows a uniform Bi_2_O_3_ NP distribution throughout the device thickness, indicating a homogeneous distribution of NPs and minimal dead volume without any NP sedimentation unlike previous reports^10^. Such distribution of the NPs offer an efficient X-ray to charge direct conversion, throughout the entirety of the device thickness. SEM and atomic force microscopy topographical analysis (Supplementary Figure 9) of the films suggests NP aggregation on the film surfaces which we have previously reported to enable efficient charge extraction through enhancements in the electric field via metal electrode structuring ^26^.

### Charge transport analysis

**Fig. 5:**
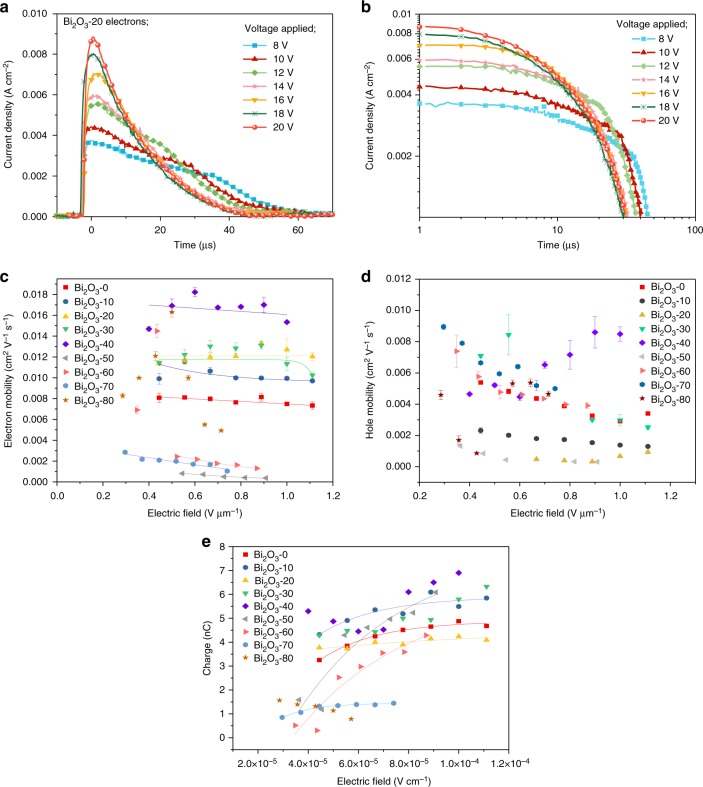
Charge transport analysis. **a** Time-of-flight transients of electrons for the Bi_2_O_3_-20 device for the applied reverse-bias voltages from 8 to 20 V. **b** The double logarithmic plot of the time of flight transient presented in **a**. Electric field dependency of **c** electron mobility (data have been fitted to show the mobility dependency under the electric field) and **d** hole mobility of devices from Bi_2_O_3_-0 to Bi_2_O_3_-80. Here, the error bars represent the range of three measurements carried out under each condition. **e** The collected electron charge for different devices based on a fit to the Hecht equation

Transport of both electrons and holes was analysed by photo-induced time-of-flight (TOF), a technique widely used to measure the charge carrier transport in various low mobility semiconductors^27^. The mobility ($\mu$) of the charges is determined from^28^ $\mu = d^2{\mathrm{/}}(t_{{\mathrm{trans}}} \times V)$ where *d* is the sample thickness and *V* is the biased voltage. Figure 5a and b gives an example of TOF characteristics for electrons in a Bi_2_O_3_-20 device. Calculation of the carrier mobilities from the TOF transients indicates balanced electron and hole mobilities of ~10^−3^ cm^2^ V^−1^s^−1^, with minor variation occurring for different electric fields (Fig. 5c, d). These values are comparable to those observed for thinner P3HT:PC_70_BM photovoltaic devices^29^. The Bi_2_O_3_-40 device exhibits the highest charge carrier mobility, which is in agreement with the high crystallinity observed in the GI-WAXS as explained previously, as well as through DSC given in Supplementary Information 8 where a high crystallinity in excess of 45% is observed.

An important point for consideration in the development of X-ray detectors is their CCE, described by the Hecht equation^14^;

2 $${ Q = Q_0\left\{ {\frac{{\mu _{\mathrm{h}}\tau _{\mathrm{h}}E}}{d}\left[ {1 - {\mathrm{exp}}\left( { - \frac{x}{{\mu _{\mathrm{h}}\tau _{\mathrm{h}}E}}} \right)} \right] + \frac{{\mu _{\mathrm{e}}\tau _{\mathrm{e}}E}}{d}\left[ {1 - {\mathrm{exp}}\left( { - \frac{{d - x}}{{\mu _{\mathrm{e}}\tau _{\mathrm{e}}E}}} \right)} \right]} \right\}}$$

Here, *Q* and *Q*_0_ are the number of charges extracted and generated respectively at a distance *x* from the anode, *E* is the electric field and *µ*_h_, *µ*_e_, *τ*_h_ and *τ*_e_ are the hole and electron mobilities and transit times, respectively. Figure 5e exhibits the collected electron charge for different devices with a fit for the Hecht equation. For the BHJ-NP detectors, the CCE of electrons and holes separately are ~63% with relatively similar $\mu \tau$ product of $\mu \tau \approx$10^−7^ cm^2^ V^−1^.

### Origins of high sensitivity

As discussed previously, a thousand-fold increase in charges extracted is observed with respect to the absorbed X-rays for Bi_2_O_3_-10 to Bi_2_O_3_-70 devices which increases to ~× 10^5^ for Bi_2_O_3_-80 devices (Fig. 2d). An important effect for enhanced sensitivity is impact ionization due to hot X-ray photoelectrons (PEs) and holes which enables ~10^3^ free carriers to be generated per photon^30^. However, the CCE of ~63% points towards the possibility to an additional mechanism for sensitivity enhancement. One contributing factor towards this enhancement is photoconductivity gain, which results from trapping either electrons or holes^14^. The emergence of rise decay times well exceeding 100 ms from the device Bi_2_O_3_-50 upwards suggests photoconducting gain as a possible sensitivity enhancement mechanism for Bi_2_O_3_-50 devices and upwards. However, the <100 ms rise times for the lower NP loading is indicative of a different gain mechanism that enables fairly high rise and decay times to be observed.

In astrophysical environments, it is well known that dust and ice NPs efficiently scatter X-rays due to their grain geometric sizes being on the same scale as the photon wavelengths^31,32^. In such studies, the classical Mie model is used as an approximation in determining differential and total scattering cross sections for nano-sized particles^32^. Mie-scattering results when electromagnetic radiation (EMR) interacts with particles with dimensions larger than the EMR wavelength. Due to the sub-nanometre wavelength of X-rays, NPs are extremely efficient Mie scatters of X-rays where the ratio of scattered X-ray intensity $I_{\mathrm{s}}\left( \lambda \right)$, to the irradiated X-ray intensity $I_{\mathrm{R}}\left( \lambda \right),$ can be found through ^33^,

3 $$\frac{{I_{\mathrm{S}}\left( \lambda \right)}}{{I_{\mathrm{R}}\left( \lambda \right)}} = {\int_{r_{\mathrm{d}}}^{R_{\mathrm{o}}}} {{\int_0^\pi} {{\int_0^{2\pi }} {\sin \theta {\mathrm{d}}\theta {\mathrm{d}}\varphi \,{\mathrm{d}}r} } } \int_{a_{{\mathrm{min}}}}^{a_{{\mathrm{max}}}} {\frac{{\partial n(r,a)}}{{\partial a}}\frac{{\left| {f\left( {\lambda ,\theta ,a} \right)} \right|}}{{1 + \frac{{r^2}}{{R^2}} - 2\frac{r}{R}}}\,{\mathrm{d}}a}$$

where *R* and *r*_d_ are the distance from the X-ray source to the detector and the detector thickness. *a* is the NP radius, $\theta$ is the X-ray scattering angle, $\varphi$ is the Azimuthal angle with respect to the axis source-detector and *λ* is the radiation wavelength. The differential cross section for the scattering of unpolarised radiation is given in Supplementary Note 4.

**Fig. 6:**
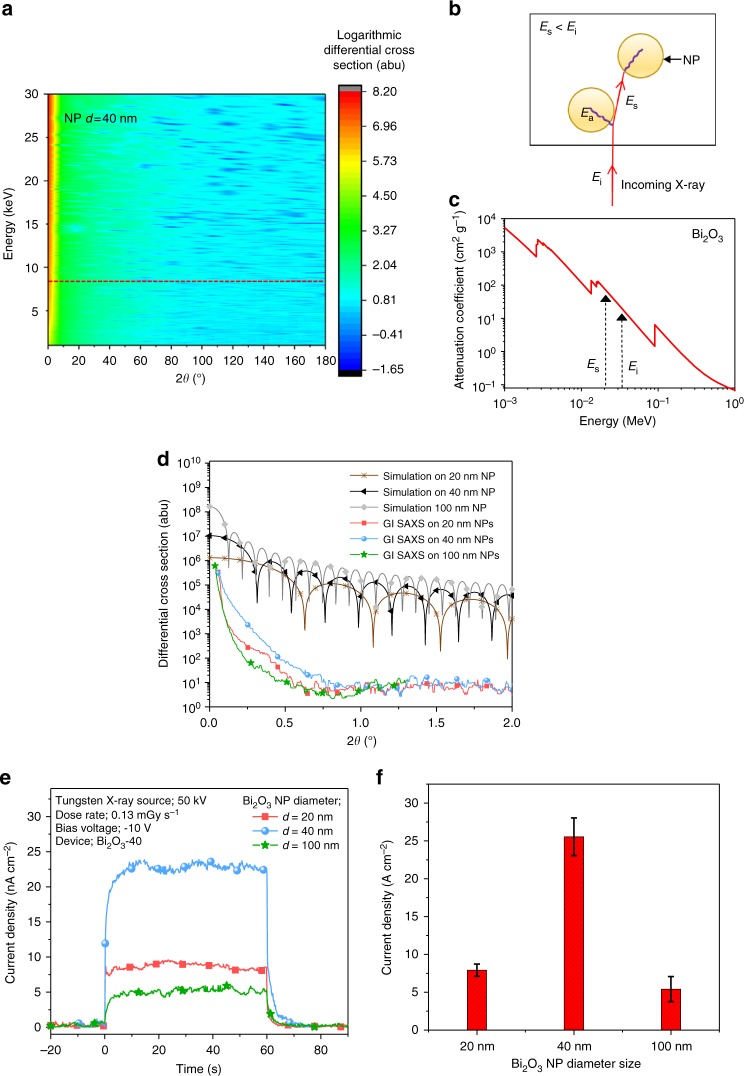
X ray scattering by nanoparticles. **a** Simulated differential cross sections for X-ray scattering from Bi_2_O_3_ NPs with a diameter (*d*) of 40 nm. **b** The schematic of the inelastic Mie-scattering mechanism and **c** the mass attenuation coefficient curve of Bi_2_O_3_. Here, *E*_i_, *E*_a_ and *E*_s_ are the energy of the incident X-ray photon, energy absorbed and the energy of the scattered photon, respectively. **d** The comparison of simulated differential cross section and extracted 1D data of experimental grazing incidence small angle X-ray scattering (GI-SAXS) at 8 keV energy for films with *d* = 20, 40 and 100 nm NPs. (The dotted line in **a** represents the extracted data at 8 keV.) **e** X-ray photocurrent response of the polymer diodes containing Bi_2_O_3_ NP sizes of 20, 40 and 100 nm and **f** the current density value with the spread for six detectors

In order to assess the impact of the NP size on the Mie scattering of X-rays, we simulated the Mie-scattering process for spherical Bi_2_O_3_ NPs with diameters (*d*) of 20, 40 and 100 nm. The NP size is crucial for detector sensitivity as NPs in the quantum dot regime (<10 nm) lead to indirect X-ray detection^34^, while large nanoparticles (≥100 nm) reduce the extraction probability for charges generated within the NP and also reduce the differential scattering cross section as the particle size becomes very much greater than the X-ray wavelength^31^. These simulations do not take into consideration the effect of an ensemble of NPs, particle size distribution of the NPs, the formation of NP clusters, or deviations in the aspect ratio from a simple spherical geometry. Figure 6a shows the angular dependent differential cross sections for photon energies ranging from 1 to 30 keV for *d* = 40 nm NPs, where a high scattering effect can be seen at small angles. Considering a simplistic scenario where the Bi_2_O_3_ NPs are arranged in a periodic structure throughout the detector volume, an incident X-ray photon (on a NP) with energy of 8 keV (*E*_i_) has a high scattering cross section within 2° (Supplementary Figure 10 and Supplementary Note 5). Under elastic scattering this photon will be scattered by ~45 NPs within the detector volume. However, as Mie scattering is an inelastic process, the scattered photon will have a lower energy (*E*_s_) than *E*_i_. Based on the X-ray attenuation curve for Bi_2_O_3_, this results in the deposition of more energy (equivalent to *E*_s_) as the scattered photon interacts with a subsequent NP (Fig. 6b, c). Therefore, tuning the NP loading such that two NP scattering sites are placed in close proximity to each other enables a significant enhancement (×100) in the X-ray path length due to the X-ray scattering process, and energy deposited even within layers that are dimensionally “thin” for X-ray detection (i.e. <100 µm).

Following the above simulations, we proceeded to confirm the predicted enhancement in X-ray scattering through Grazing Incidence-Small Angle X-ray Scattering (GI-SAXS) for BHJ-Bi_2_O_3_ NP films (same composition) consisting of three different NP sizes (*d* = 20, 40 and 100 nm) (characterization of the Bi_2_O_3_ NPs used in this study is given in Supplementary Figure 11a, c and Supplementary Note 6). The comparison between the simulation and experimental scattering (at 8 keV) is given in Fig. 6d. The GI-SAXS results are in good agreement with the predictions based on the Mie-scattering effects, with approximately an order of magnitude difference in X-ray scattering at angles < 0.75° for the *d* = 20 and 40 nm NP dimensions. However, as the NP diameter increases, up to 100 nm, the X-ray scattering is reduced by two orders of magnitude as compared to the *d* = 40 nm device. While this observation is not supported by the *d* = 100 nm simulation carried out for a single sphere, GI-SAXS analysis (Supplementary Figure 11b) indicates that the X-ray scattering in this film takes place from ~ 15 nm sized features. This is indicative of the importance of optimum NP packing in these films to achieve the desired Mie-scattering effects.

In order to assess the impact of the Mie-scattering process on the detector sensitivity, X-ray detectors were fabricated using the three different NP sizes (*d* = 20, 40 and 100 nm) under the Bi_2_O_3_-40 condition, and tested under a 50 kV X-ray source (Fig. 6e, f). The *d* = 40 nm NP detectors show a three times higher response compared to the *d* = 20 and 100 nm NP devices, which is in good agreement with the enhanced X-ray scattering theoretically predicted for the *d* = 40 nm NPs. We note that while quantum mechanical effects due to nanoscopic features can also influence the scattering processes this would require more in-depth theoretical and experimental studies, which are outside the scope of this work.

## Discussion

In conclusion, we have developed a hybrid ‘inorganic in organic’, direct transduction X-ray detector that delivers outstanding sensitivities of 1712 and 58 µC mGy^−1^ cm^−3^ for soft and hard X-rays respectively. Furthermore, flexible detectors also show a high sensitivity approaching 300 µC mGy^−1^ cm^−3^, highlighting the promise of the technology for dosimetry and imaging in non-planar architectures. The improved X-ray sensitivity is a result of impact ionization, and an enhanced path length due to Mie scattering and the efficient separation, and transport of these by the BHJ-NP architecture resulting in high-charge collection efficiencies (>60%). Based on this concept, a preliminary flat panel imager has also been demonstrated. The method of direct detection and imaging, combined with low cost, flexibility and scalability for large-area manufacture, improves on current solid state X-ray detector performance by 2–3 orders of magnitude, under low voltages, while delivering novel attributes suitable for a range of current and previously unexplored detection and imaging applications.

## Methods

### Materials

Regioregular poly(3-hexylthiophene-2,5-diyl) (P3HT, 40 mg, Rieke 4002 EE) and [6,6]-Phenyl C_71_ butyric acid methyl ester (PC_70_BM, 40 mg, 99% pure; Solenne) were added to dichlorobenzene (1 ml) to produce a P3HT:PC_70_BM (Bi_2_O_3_-0) solution. Bi_2_O_3_ nanoparticles (β phase with a tetragonal crystal structure; 38 nm diameter; surface area 18 m^2^ g^−1^; Alfa Aesar) were dispersed in P3HT:PC_70_BM solution to give Bi_2_O_3_ concentrations of 10 (Bi_2_O_3_-10), 20 (Bi_2_O_3_-20), 30 (Bi_2_O_3_-30), 40 (Bi_2_O_3_-40), 50 (Bi_2_O_3_-50), 60 (Bi_2_O_3_-60), 70 (Bi_2_O_3_-70) and 80 (Bi_2_O_3_-80) mg ml^−1^.

### Device fabrication

Rigid devices were fabricated on ITO (In_2_O_3_:Sn) glass substrate (15 mm × 15 mm, 10 Ω per square, Luminescence Technology Corp.) and flexible devices were fabricated on Kapton substrates (15 mm × 15 mm, Dupont), with a patterned chromium (Cr; 10 nm)/gold (Au; 50 nm) bilayer contact deposited as the anode using e-beam evaporation. A electron blocking and hole transporting Poly(3,4-ethylenedioxythiophene)-poly(styrenesulfonate) (PEDOT:PSS; P VP Al 4083; Heraeus) layer was spin coated in air (5000 rpm for 40 s) and annealed at 150 °C for 10 min to give a thickness of 40 nm. Bi_2_O_3_ solutions (90 µl) were then casted. Devices were annealed (60 °C) for 20–45 min in air, until a relatively dry layer was obtained. After the low temperature annealing process, all the devices were annealed at 140 °C for 10 min in a N_2_ glove box (MBraun MB20G). Devices were kept under vacuum at a pressure of less than 3 × 10^−6^ mbar for 24 h to remove any residual solvent. This was followed by deposition of the hole blocking layer (5 nm thickness), 1-2,9-dimethyl-4,7-diphenyl-1,10-phenanthroline (BCP; sublimed grade, Sigma Aldrich, 99.99% purity) followed by deposition of an Al cathode (~150 nm) by thermal evaporation. Device encapsulation was carried out with the deposition of UV light cure adhesive glue (20 µl of Ossila Ltd) pressed with an encapsulation glass slide (Ossila) for the rigid devices, and a Kapton sheet for the flexible devices and the epoxy UV cured for 5 min.

### Electrical characterization

All the measurements are carried out in air at room temperature using an active device area of 0.68 cm^2^. A Keithley 2400 source measure unit was used to measure the current-voltage characteristics. The photocurrent performances of the devices were characterised in air using a 150 W Xe arc lamp solar simulator (Abet Technologies) fitted with an AM 1.5G filter. A reference Si cell (Newport, PVM 165) was used to adjust the intensity of the lamp to 100 mW cm^−2^.

### X-ray irradiation and characterization

Six devices were tested for each X-ray irradiation measurement. Three different X-ray beam sources were employed for the characterisation of the detectors:

1. A tungsten tube X-ray beam (Seifert RP-149 Semiconductor Irradiation System) with accelerating voltage of 50 kV under a dose-rate range of 27–131 µGy s^−1^. Dose calibration was completed using an ion beam chamber (Radcal; 10 × 6–6). A Keithley 2400 source measure unit was used for recording the electrical characteristics.

2. A 70 kV X-ray source (Siemens MEGALIX Cat Plus 125/40/90, 124GW) with a tungsten anode. The X-ray spectrum was filtered with a 2.5-mm-thick Al plate.

3. 6 and 15 MV X-rays from a multi-mode linear accelerator (Clinac iX, Varian USA) located at the Royal Surrey County Hospital. Dose rates from 100 to 400 cGy min^−1^ were provided by the LINAC and a Keithley 2400 source measurement unit was used for recording the electrical characteristics of the devices.

### Image readout and processing

An X-ray imager was fabricated using a method reported elsewhere^7^ under the device architecture of ITO/PEDOT:PSS/P3HT:PC_70_BM:Bi_2_O_3_/Au. The P3HT:PC_70_BM:Bi_2_O_3_ ratio is 1:1:1 (equivalent to Bi_2_O_3_-40 device) with an active layer thickness of 250 µm.

X-ray beam source (2) was used for the characterisation.

Images were taken by custom-made driving and readout electronics with a commercial available readout IC (ROIC) (ISC9717 from Flir). The backplane of the imager consists of a Borosilicate glass substrate with an array of 256 × 256 a-Si:H TFTs with 98 μm pixel pitch. The signal at the input was simultaneously integrated, amplified, low pass filtered and converted from analog to digital with a 14-bit converter. The integration time was 10 ms and the integrator feedback capacitance *C*_f_ was 4 pF. To eliminate fixed pattern noise and image inhomogeneity, a flat fielding has been performed.

### GI-SAXS

Devices for GI-SAXS were fabricated as stated above, but with 20, 38 and 100 nm Bi_2_O_3_ NPs in Bi_2_O_3_-40 devices. Measurements were completed on a XEUSS 2.0 (Xenocs, France) equipped with a Cu *K*_*α*_ microfocus source and a Pilatus 300k detector (Dectris, Switzerland). The scattering vector (*q*) range of the data was calibrated using a silver behenate standard material. The detector was positioned 2.495 m away from the sample to utilise the lowest *q* range available. The sample was aligned such that the surface was in the centre of the beam and parallel with the beam. For the GI-SAXS measurements, the sample was tilted by 1°. This moved the specular reflection sufficiently far away in *q* that the scattering from the particles on the surface could be measured. The scattering was recorded for 30 min.

### GI-WAXS

X-ray measurements were performed with a Xeuss 2.0 (XENOCS, France) system. The system is equipped with a MetalJet (Excillum, Sweden) liquid gallium source, providing a 9.24 keV X-ray beam collimated to a beam spot of 400 µm laterally at the sample position, measuring the full sample length. X-ray diffraction patterns were acquired with a Pilatus3R 1M 2D detector (Dectris, Switzerland) placed at ~311 mm from the sample. The distance between the sample and the detector was measured using a silver behenate calibrant in transmission geometry. Samples were measured in GI-WAXS geometry at an incident angle of 0.3° (calculated to probe the entire film thickness) under vacuum atmosphere. Diffraction images were then remapped from pixel to scattering vector coordinates with the calibration equations reported^35^ and by using MATLAB software.

### SEM/AFM/EDX

SEM was carried out using a FEI Quanta 200F Environmental scanning electron microscope and AFM was carried out using a VEECO Dimension 3000 atomic force microscope. EDX analysis was carried out using an x-act Oxford Instrument system coupled with the SEM.

### Time-of-flight

Devices were fabricated as stated above, but with a 20 nm thick Al contact for irradiation through the Al (hole transport analysis). As an excitation source, a 6 ns-pulsed Nd:YAG laser (Quantel, 532 nm, 45 mJ) was used, and the bias voltage was applied to the sample using a Keithley 2400. The transient current was measured as the voltage drop over a load resistor (10 kΩ) and recorded with an oscilloscope (Agilent infiniium 1 GHz, 4 GSa s^−1^)

### Simulation

FLUKA, a Monte Carlo simulation programme designed for the interaction and transport of articles and nuclei in matter, was used for simulation of the X-rays interaction with the active material. In order to simulate the energy depletion of our devices, the geometry of the P3HT:PC_70_BM BHJ with embedded Bi_2_O_3_ NPs (the thickness variation of the active layer was obtained experimentally) were modelled and irradiated with 50 kV X-ray photons to be processed by the FLUKA programme. Mie-scattering differential cross section simulations were carried out using MATLAB software, using a Mie function.

### Data availability

The data supporting the findings of this study is available at 6571337.

## Electronic supplementary material

Supplementary Information

Supplementary Information
