# Supplementary material for: High sensitivity organic inorganic hybrid X-ray detectors with direct transduction and broadband response
Source: Nat Commun. 2018 Jul 26;9:2926. doi: 10.1038/s41467-018-05301-6 (PMC6062530; doi:10.1038/s41467-018-05301-6)
Supplement: Supplementary file 1 — Supplementary Information [file 41467_2018_5301_MOESM1_ESM.pdf]

SUPPLEMENTARY INFORMATION

High sensitivity organic inorganic hybrid X ray detectors with direct transduction and broadband response

Thirimanne et al.

Supplementary Figures

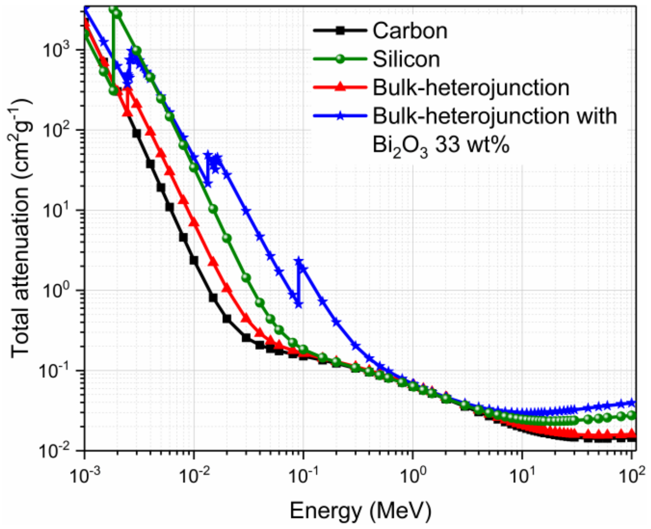

Supplementary Figure 1. X-ray mass attenuation coefficients Total attenuation of X-rays for different materials as a function of energy. Data have been calculated using NIST-XCOM software (http://www.nist.gov/pml/data/xcom/).

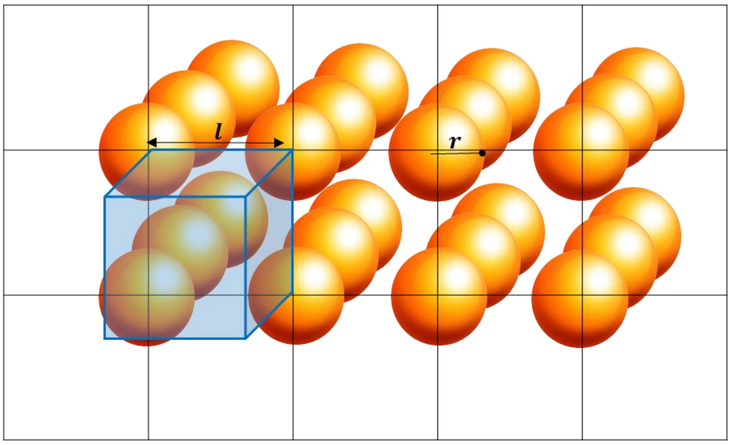

Supplementary Figure 2. Unit cell calculation The calculation of the parameters of a unit cell is presented in Supplementary Note 1.

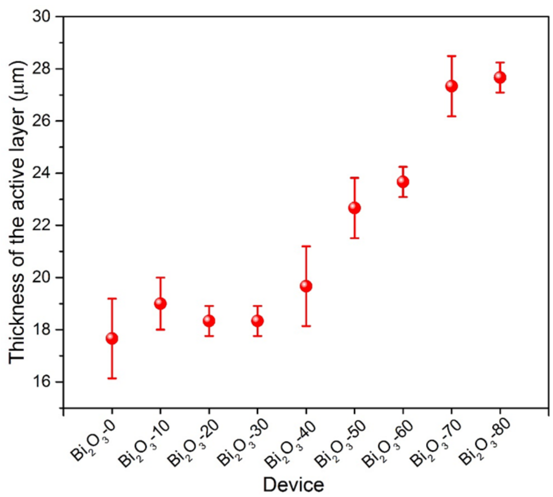

Supplementary Figure 3. Thickness variation Thickness variation of the active layer of devices with increasing Bi_2O_3 NP loading. The error bars represent the spread of the thicknesses of 4 films in each condition.

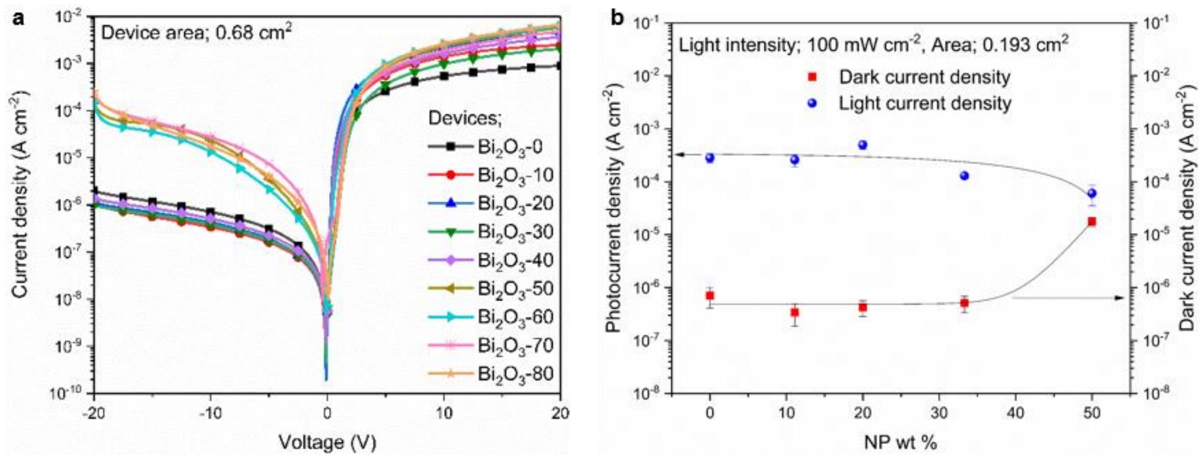

Supplementary Figure 4. Dark current and photocurrent analysis (a) Dark current density-voltage response for different Bi_2O_3 loadings. (b) Dark current density and photocurrent density of different NP loaded devices when operated at -10 V external bias voltage (the fitted lines are added as a guide to the eye). The average and the spread for three devices per NP loading is indicated by error bars. A light intensity of 100 mW cm^{-2} used to illuminate an active area of 0.193 cm^2 to obtain the photocurrent response.

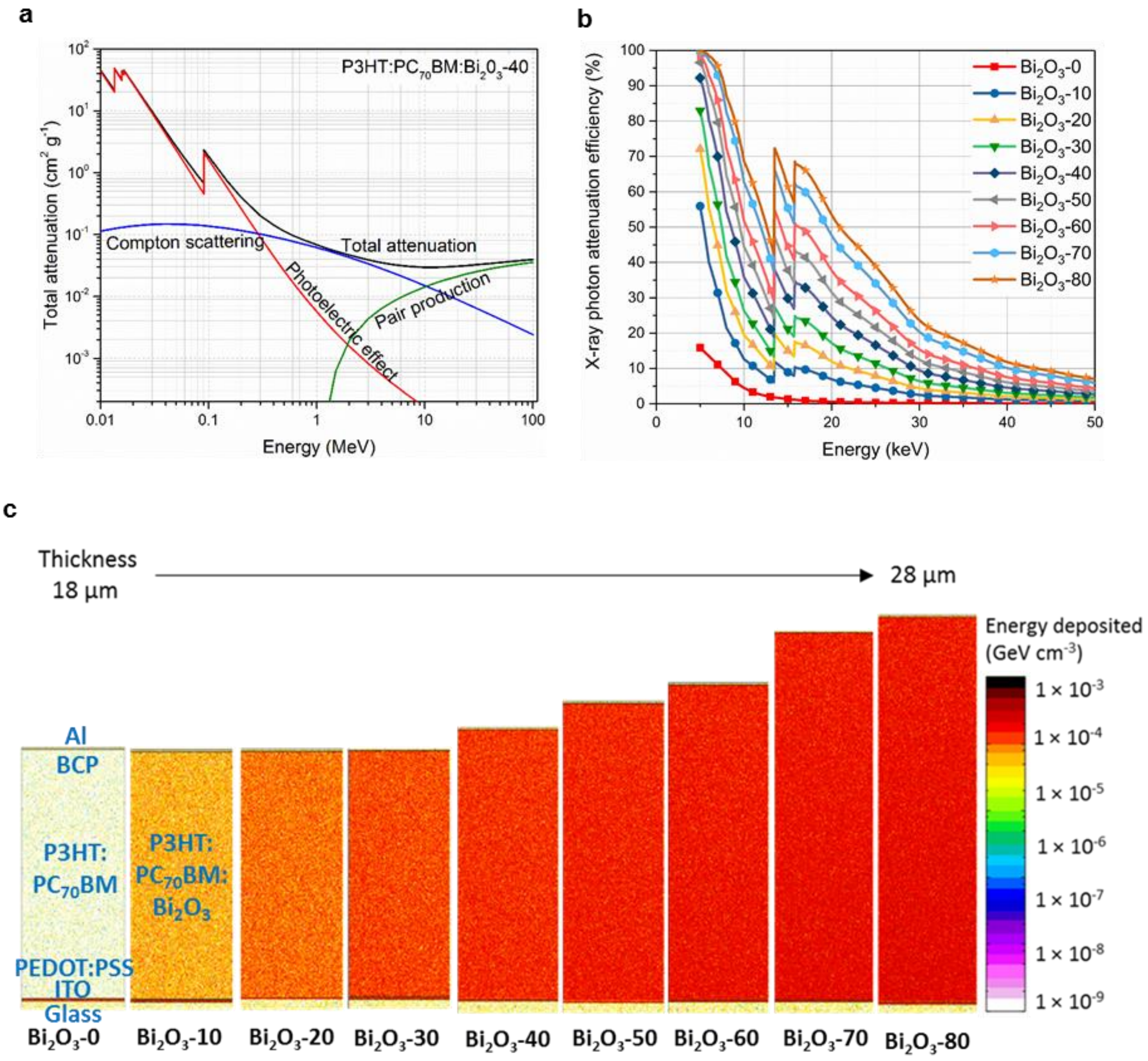

Supplementary Figure 5. X-ray interaction with matter (a) Total mass attenuation of X-rays by Bi_2O_3-40 device showing the contribution from photoelectric effect, Compton scattering and pair production, as a function of energy. Data have been calculated using NIST-XCOM software (http://www.nist.gov/pml/data/xcom/). (b) X-ray photon attenuation efficiency (using a W X-ray source). (c) Energy deposited in Bi_2O_3-0 to Bi_2O_3-80 devices simulated using FLUKA software (http://www.fluka.org/fluka.php). Here, P3HT and $PC_{70}BM$ are regioregular poly(3-hexylthiophene-2,5-diyl) and [6,6]-Phenyl C_{71} butyric acid methyl ester respectively.

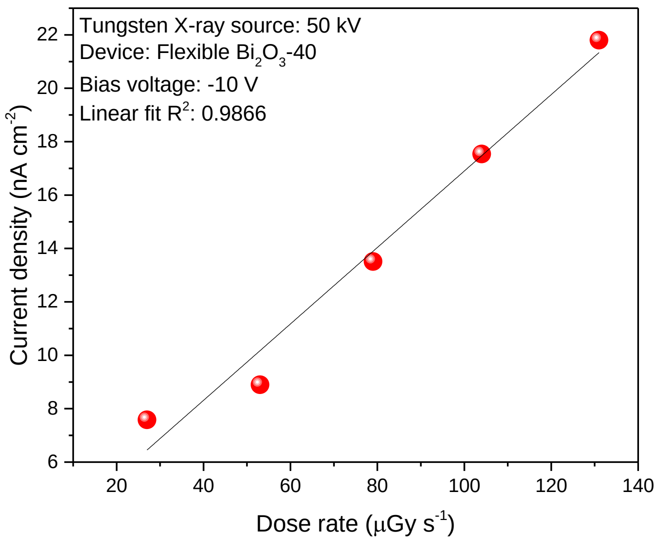

Supplementary Figure 6. Dose rate dependency Performance of the flexible Bi_2O_3-40 device under different dose rates at -10 V.

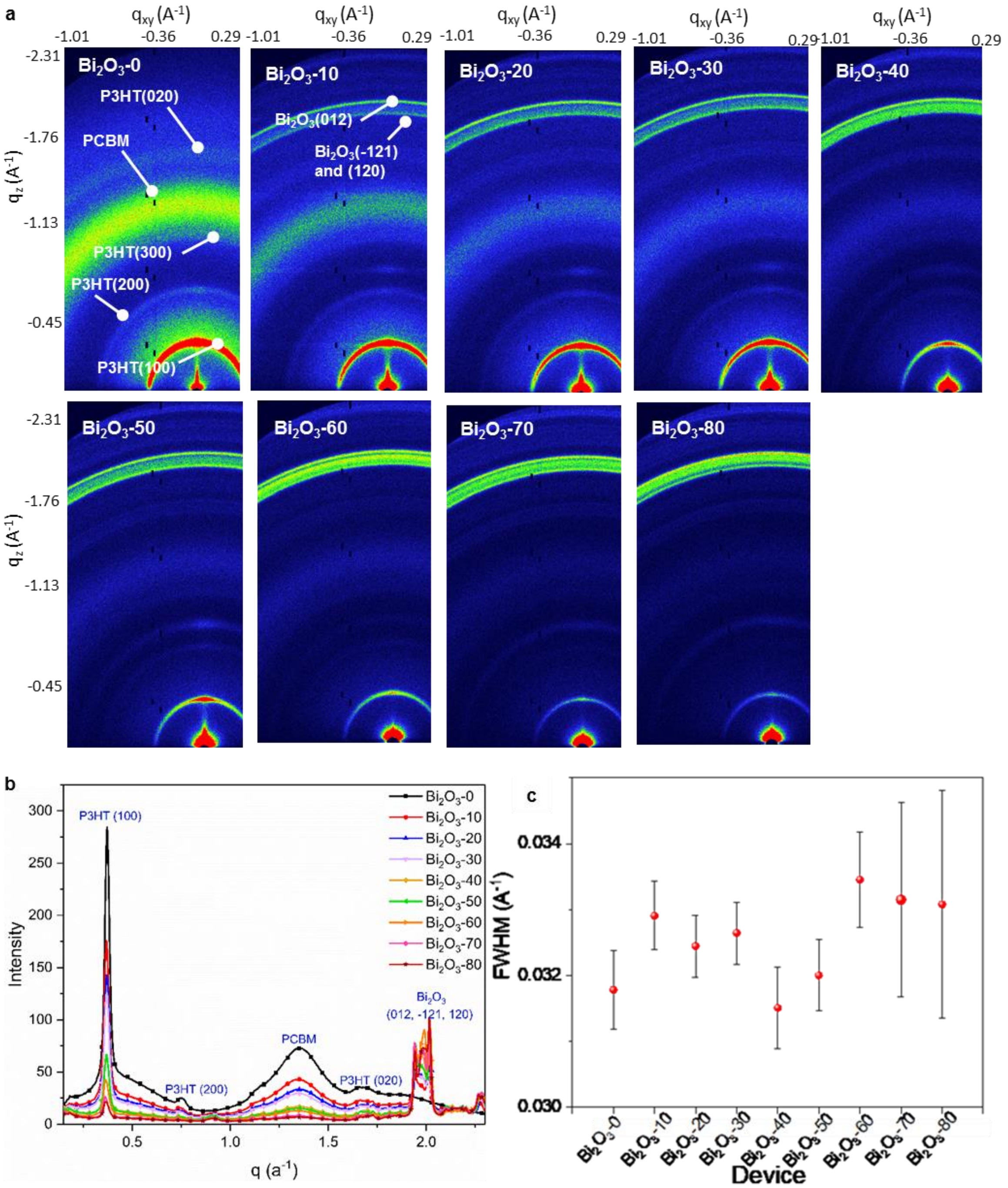

Supplementary Figure 7. Grazing incidence wide angle X-ray scattering data (a) A comparison of (grazing incidence wide angle X-ray scattering) GI-WAXS 2d detector data for all the devices from Bi_2O_3-0 to Bi_2O_3-80 nanoparticle loading composition. (b) GI-WAXS data represented as a 1D plot and (c) the full width at half maximum (FWHM) of P3HT (100) peak. The error bar represents error of the fit of calculating FWHM. Further discussion on the GI-WAXS data is given in Supplementary Note 2.

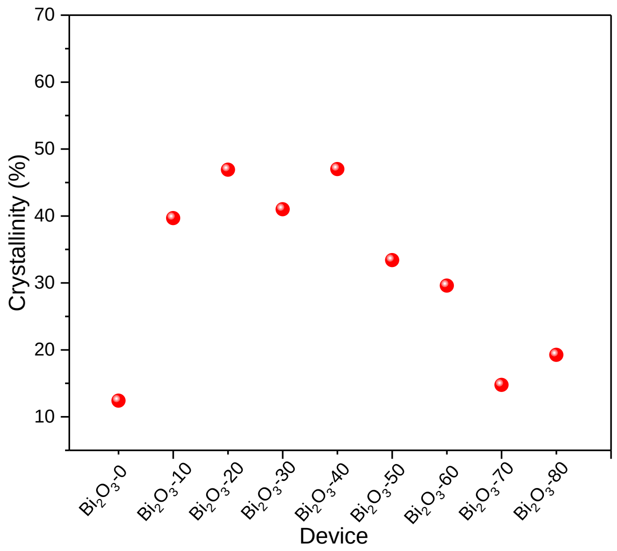

Supplementary Figure 8. Material crystallinity Crystallinity of the device layers calculated using differential scanning calorimetry (DSC). Further analysis on the crystallinity is presented in Supplementary Note 3.

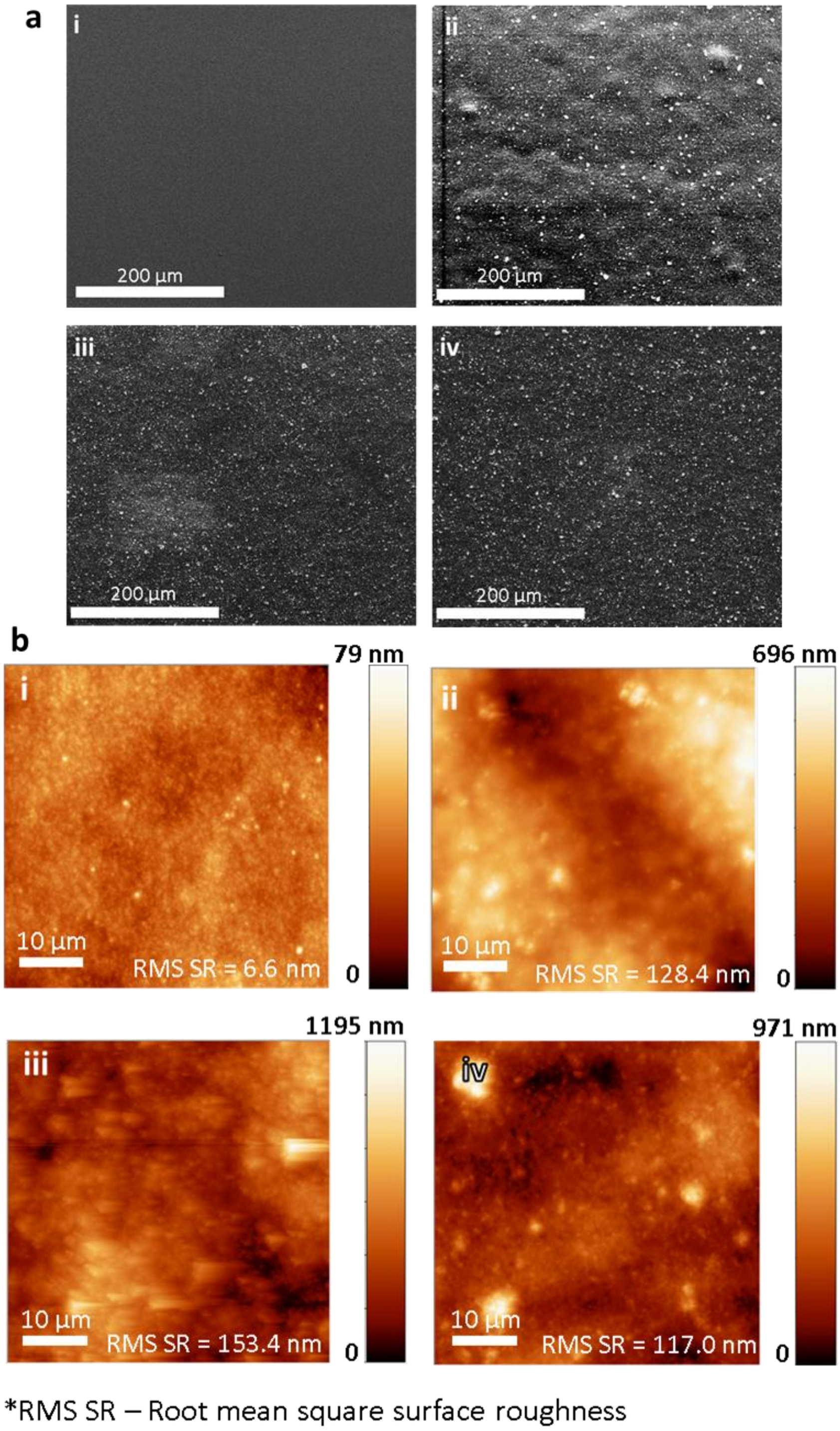

Supplementary Figure 9. Surface analysis (a) Scanning electron microscopy (SEM) and (b) Atomic force microscopy (AFM) images of the surface of the P3HT:$PC_{70}BM$ active layers, for (i) Bi_2O_3-0, and with different Bi_2O_3 concentrations of (ii-iv) Bi_2O_3-20, Bi_2O_3-40, and Bi_2O_3-80. The images indicate NP texturing of the surface upon the incorporation of Bi_2O_3 into the organic semiconductor system. The root mean square (RMS) surface roughness (SR) values are given under each AFM image.

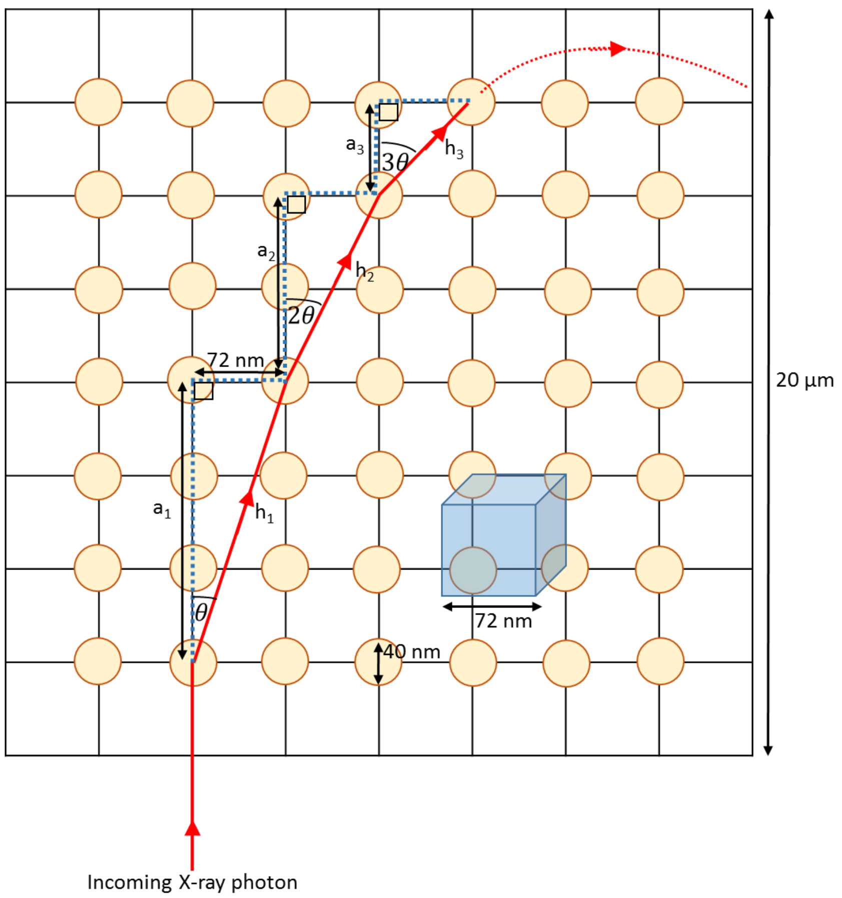

Supplementary Figure 10. Scattering model Scattering of an incident X-ray photon due to multiple nanoparticles. The mechanism and the analysis of the scattering model is presented in Supplementary Note 5.

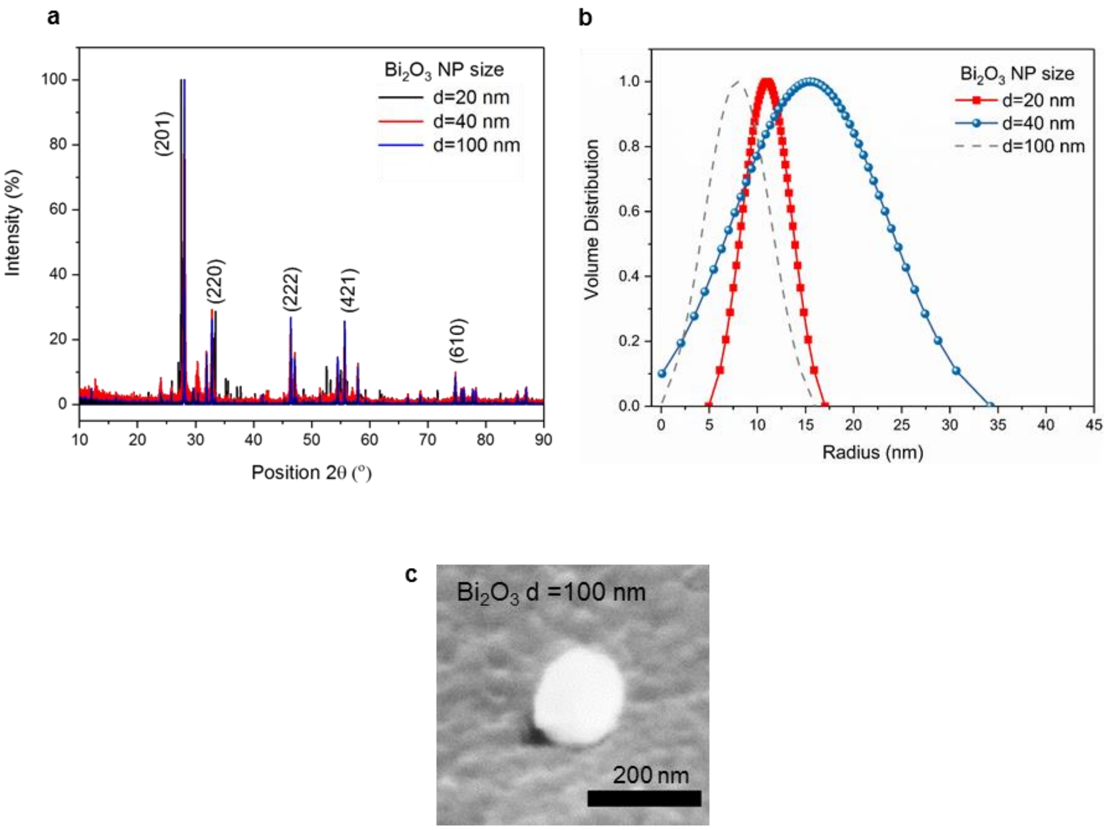

Supplementary Figure 11. Characterisation of nanoparticles. (a) X-ray diffraction (XRD) pattern of Bi_2O_3 nanoparticles (NPs) (with diameters (*d*) of 20, 40 and 100 nm) (b) Normalised Gaussian distributions of the measured particle radii using (grazing incidence small angle X-ray scattering) GI-SAXS, for the Bi_2O_3 NPs in the active volume of the device, and (c) an SEM image of a 100 nm particle. Further discussion on the characterisation of NPs is presented in Supplementary Note 6.

Supplementary Notes

Supplementary Note 1: Unit cell calculation

Assuming a uniform distribution of spherical Bi_2O_3 nanoparticles, the distance from the centre of one NP to the adjacent NP (l), can be calculated as follows;

Radius of the NP (nm) – r

Loading of NP in the device – W

Density of Bi_2O_3 – ρ

Area of the device – A

Thickness of the device - d

Length of a unit cell - l

Volume of a NP = $\frac{4}{3}\pi r^3$

Assuming one unit cell is occupied by one NP;

Total number of NPs = Total number of unit cells = $\frac{W}{\left(\frac{4}{3}\pi r^3\right) \times \rho}$

Total volume of unit cells = $A \times d$

Total volume of NPs = $\frac{W}{\left(\frac{4}{3}\pi r^3\right) \times \rho} \times l^3$

$$A \times d = \frac{W}{\left(\frac{4}{3}\pi r^3\right) \times \rho} \times l^3 \quad (1)$$

$$l = \sqrt[3]{\frac{(A \times d)}{W} \times \frac{4}{3} \times \pi r^3 \rho} \quad (2)$$

Supplementary Note 2: Analysis of the grazing incidence wide angle X-ray scattering

The first P3HT reflection (100) at q ≈ 0.36 $Å^{-1}$ indicates a large spread of orientations between edge-on and face-on lamellae with a slight predominance of edge-on lamellae. $PC_{70}BM$ does not appear to assume any preferential orientation with respect to the substrate, as evident from the amorphous ring at q ≈ 0.62 $Å^{-1}$ [1,2,3]. Adding Bi_2O_3 leads to extra diffraction rings appearing, with the broad ring between q ≈ 1.61 and 1.65 $Å^{-1}$ being the (-121), (120) and (012) Bi_2O_3 reflections.[4]

To gain further understanding of the impact of NP addition on the crystallinity of the P3HT phase, 1D-GI-WAXS (Supplementary Figure 7b) was analysed. The width of the P3HT (100) X-ray peak (full width at half maximum (FWHM)) (Supplementary Figure 7c) in the q direction is correlated with the crystallinity of the material and related to the grain size through Scherrer's equation[5].

Supplementary Note 3: Crystallinity using differential scanning calorimetry.

In order to independently confirm the crystallinity, differential scanning calorimetry (DSC) was carried out on the deposited films, where again the highest crystallinity (in the range of 40-50 %) was observed for Bi_2O_3-10 to Bi_2O_3-40 films, in agreement with the conclusions made from the GI-WAXS measurements (Supplementary Figure 7). The high crystallinity results in lower trap states, enabling low rise and decay time constants observed for Bi_2O_3-0 to Bi_2O_3-40 devices under X-ray irradiation (Figure 2g).

Supplementary Note 4: Mie scattering of X-rays by nanoparticles

The differential cross section for the scattering of unpolarised radiation can be written as;

$$|f(\lambda, \theta|^2 = \frac{\lambda^2}{8\pi^2} (|S1\ (\theta)|^2 + |S2\ (\theta)|^2) \tag{3}$$

Where, λ is the radiation wavelength and θ is the X-ray scattering angle. The complex scattering amplitudes $S1\ (\theta)$ and $S2\ (\theta)$ are defined as;

$$S1\ (\theta) = \sum_{n=1}^{\infty} \frac{2n+1}{n(n=1)} [a_n \pi_n(cos\theta) + b_n \tau_n(cos\theta)] \tag{4}$$

$$S2\ (\theta) = \sum_{n=1}^{\infty} \frac{2n+1}{n(n=1)} [b_n \pi_n(cos\theta) + a_n \tau_n(cos\theta)] \tag{5}$$

Where the angular fractions $\pi_n(cos\theta)$ and $\tau_n(cos\theta)$ are defined as;

$\pi_n(cos\theta) = \frac{1}{sin\theta} P_n^1(cos\theta)$ and $\tau_n(cos\theta) = \frac{d}{d\theta} P_n^1(cos\theta)$

With $P_n^m\ (cos\theta)$ being the associated Legendre polynomials.

Supplementary Note 5: Scattering model

The following scattering model is explained assuming a primitive cubic lattice of uniform spherical Bi_2O_3 NPs distributed through-out the active layer of the device. The NPs are considered as points in each unit cell.

The example shows Bi_2O_3-40 device (with an active layer thickness of 20 µm), where the unit cell length is 72 nm. As the simplest scenario, we have considered an X-ray photon irradiating a NP in the first unit cell row, and being scattered away on one direction only. θ is the scattered angle with respect to the incoming X-ray. Using the Mie scattering simulation (Figure 6 a and d), θ = 2°. Hence, the path which the X-ray photon travels prior to interacting with the second NP is h_1 and this corresponds to vertical device length (a_1 = 2061 nm). The X-ray will be scattered from each colliding NP, while reducing the X-ray travel length (a_2 = 1029 nm, a_3 = 685 nm, a_4 = 512 nm and so on). Under the assumption that the X-ray energy does not dissipate, at the point where the X-ray angle reaches 90° (corresponds to 45 scattering sites), the sum of the X-ray travel length ($\sum a$) equals to 8110 nm, within the device thickness of ~20 µm fabricated in this work.

Hence, in the real scenario, considering N number of randomly placed NPs, with each having scattering in many angles, there is a possibility of having three orders of magnitude enhancement of charges within the bulk material itself.

Supplementary Note 6: Characterisation of nanoparticles

Bi_2O_3 NPs with diameters of 20, 40 and 100 nm were subjected to X-ray diffraction (XRD) (Supplementary Figure 11a). All the recorded peaks are in good agreement with the literature data.

Active layers consisting of the Bi_2O_3 NP sizes (20, 40 and 100 nm) were subjected to GI-SAXS to obtain a NP radii distribution. For the 20 and 40 nm samples, the 1D-SAXS signal give radii in the correct q region equivalent to the corresponding NP diameters. Hence, the layers contain NPs with an average expected radii, without forming clusters or aggregates.

In the device with the 100 nm diameter NPs, the q region is closer to the beamstop, hence we are unable to make a correction for the beam related effects around the beamstop. There is a region at higher q values that is indicative of scattering from voids in-between the larger particles (the distance between two NPs). This has been modelled using a sphere, similar to the other samples, which produces a distance of 15.92 nm. In order to independently verify the particle size of the NPs used, an SEM analysis was carried out which indicates the average particle size to be ~ 100 nm (the inset shows an SEM image of representative NP).

Supplementary References

1. Lilliu, S. *et al.* Dynamics of Crystallization and Disorder during Annealing of P3HT/PCBM Bulk Heterojunctions. *Macromolecules* **44,** 2725–2734 (2011).

2. Lilliu, S. *et al.* Absence of Structural Impact of Noble Nanoparticles on P3HT:PCBM Blends for Plasmon-Enhanced Bulk-Heterojunction Organic Solar Cells Probed by Synchrotron GI-XRD. *Sci. Rep.* **5,** 10633 (2015).

3. Chu, C.-W. *et al.* Control of the nanoscale crystallinity and phase separation in polymer solar cells. *Appl. Phys. Lett.* **92,** 103306 (2008).

4. Malmros, G. *et al.* The Crystal Structure of alpha-Bi2O2. *Acta Chem. Scand.* **24,** 384–396 (1970).

5. Thermal annealing effect on the nanomechanical properties and structure of P3HT:PCBM thin films. *Thin Solid Films* **519,** 4105–4109 (2011).
