# Supplementary material for: High sensitivity organic inorganic hybrid X-ray detectors with direct transduction and broadband response
Source: Nat Commun. 2018 Jul 26;9:2926. doi: 10.1038/s41467-018-05301-6 (PMC6062530; doi:10.1038/s41467-018-05301-6)
Supplement: Supplementary file 2 — Supplementary Information [file 41467_2018_5301_MOESM2_ESM.pdf]

Reviewers' comments:

Reviewer #1 (Remarks to the Author):

The authors present an original study on direct X-ray detection processes in high-Z NPs-loaded organic BHJ diodes. In order to grant the publication of the manuscript in Nature Comm., the following points should be addressed:

1)when discussing the performance of the device, especially in terms of sensitivity, it would be useful to benchmark the reader as to what is the current state-of-the-art and with what type of technology it is reached. A wealth of papers came out in the last couple of years on this topic. In case of inorganic detectors and indirect detectors, since the presented references have been published long ago, it is necessary to replace them with recently published references and suggested sensitivity values.

2)the authors should describe in the manuscript why the authors selected the P3HT and PC70BM from various p-type and n-type polymers. P3HT and PC70PM are the most extensively used and best understood materials to form BHJ in OPV research. However, considering the purpose of improving the sensitivity, other polymers should be considered. And also describe why the authors selected the Bi2O3 NPs among various metal-oxide NPs.

3)at page 3, line 57
an additional explanation is needed to see how the value of ~ 200 V/um was came from.

4)the authors need to explain why the sensitivty was improved compared to the reference below.
A. Ciavatti et al. Dynamics of direct X-ray detection processes in high-Z Bi2O3 nanoparticles-loaded PFO polymer-based diodes. Appl. Phys. Lett. 111, 183301 (2017)

5)at page 7, lines 127-134
PC70BM is not suitable acceptor for fabricating a flexible detector. If you consider the thermal and mechanical stability, it is better to choose from non-fullerene acceptors. Probably, the sensitivity of the proposed detector was decreased after undergoing 10 cycles of ~ 3 mm curvature. Please refer to the following references.
T. Kim et al. Flexible, highly efficient all-polymer solar cells. Nat. Comm. 6, 8547 (2015)

6) in Figure 5
Bi2O3-40 showed relatively high carrier mobility. the authors need to explain the reason.

7) please provide a title that more accurately. It is better to add 'Bi2O3' and 'P3HT:PC70BM'.

Reviewer #2 (Remarks to the Author):

The authors report organic-inorganic hybrid X-ray detectors consisting of bismuth oxide nanoparticles embedded in an organic bulk heterojunction. Although many research groups have reported hybrid X-ray sensors for various applications, hybrid X-ray sensors satisfying high-sensitivity and broad-band detection have been rarely reported. The main technical comments are as follows:

1. There are many parameters to calculate the X-ray sensitivity. The parameter values for the calculated sensitivities in this work should be further justified.

2. In Fig 2(c), the measured sensitivity increases with increasing bismuth oxide NP loading. Does this mean that the sensitivity of the X-ray detector would increase with more loading of bismuth oxide NPs? In addition, is this behavior consistent with the theory?

3. EDX imaging in Fig 4(b) looks to be uniform in a macroscopic large scale. How about the other samples containing more Bi2O3 NP loading? Real distribution of NPs may be non-uniform in a microscopic scale. Additional data to confirm the uniform distribution of Bi2O3 NPs are useful and necessary.

4. In general, one of main problems for organic devices is their bad stability. How about the X-ray sensors in this work? Have you checked the stability of the X-ray sensors used in this work?

5. Which sample is used for performance comparison shown in Fig 1(a)? is the same sample used to check good broad-band detection?

6. The authors mention that the experimental and theoretical data in Fig 6(c) are consistent only with an order of magnitude difference. But, I see the difference is much bigger.

7. Is there a relation between sensitivity and broad band detection in this sample?.

Minor comments:

1. The photos in Fig 2(f) are unclear.

2. Explain solid lines in Figures (for example, Fig 5 (b,d))

Submission of revised manuscript (NCOMMS-18-07706-A): ***"High sensitivity organic-inorganic hybrid X-ray detectors with direct transduction and broadband response".***

We are grateful to the two reviewers for their comments, which we believe have improved the context, clarity and quality of the manuscript.

The points raised by the two reviewers are addressed in full below. These changes have also been highlighted in yellow in the revised manuscript submitted with this letter.

Reviewer #1 (R1):

R1 comment 1

"When discussing the performance of the device, especially in terms of sensitivity, it would be useful to benchmark the reader as to what is the current state-of-the-art and with what type of technology it is reached. A wealth of papers came out in the last couple of years on this topic. In case of inorganic detectors and indirect detectors, since the presented references have been published long ago, it is necessary to replace them with recently published references and suggested sensitivity values."

We agree with the reviewer that the benchmarking of the detector sensitivity is crucial, especially considering the developments that have taken place over the recent years. As such we have now added recently published inorganic detectors (21)[31] - (2017), (22)[32] - (2014) and (10)[12]– (2015), (23)[34] – (2017) as indirect detectors, as well as (24)[28] – (2017) for direct organic detectors with the respective sensitivity values in Figure 1 (page 4 lines 75 and 76). In addition, we would like to note the following recently published references for direct organic detectors were already in the submitted manuscript;

(1)[13]-(2016), (7)[8]-(2016), (9)[11]-(2016), (15)[23]-(2016), (16)[7]-(2017), (17)[24]-(2017), (18)[25]- (2017), (19)[26] -(2017) and (20)[27]-(2017).

R1 comment 2

"The authors should describe in the manuscript why the authors selected the P3HT andPC70BM from various p-type and n-type polymers. P3HT and PC70PM are the most extensively used and best understood materials to form BHJ in OPV research. However, considering the purpose of improving the sensitivity, other polymers should be considered. And also describe why the authors selected the Bi2O3 NPs among various metal-oxide NPs."

We have added the following section to the manuscript (lines 54 – 66) to justify the choice of the high Z material and the organic semiconductors used.

"We have chosen Bi_2O_3 from the many metal oxides available based on its direct conversion of X-rays and lower environmental impact and health risks when compared to, for example, high Z Pb based semiconductors. Given its existing use as a non-toxic dental material such as in the case of hydraulic silicate cements[15] with an opacity to X-rays makes it an ideal candidate for our application. Regioregular poly(3-hexylthiophene-2,5-diyl) (P3HT) and [6,6]-Phenyl C_{71} butyric

acid methyl ester ($PC_{70}BM$) were selected as the BHJ system. The formation of nanoscale in such BHJ diodes throughout the volume of the device, in close proximity to the NPs leads to an in-built depletion region, with local electric fields as high as ~200 V μm^{-1},[16]which has been experimentally quantified with Fourier-Transform IR-absorption spectroscopy for the P3HT:PCBM system. This is further enhanced by dielectric inhomogeneities in the material.[17,18] The above factors, in combination with the high crystallinity of P3HT:$PC_{70}BM$ enables efficient electron and hole extraction from the entirety of the depleted active layer under low reverse bias voltages (<10 V).”

R1 comment 3

“At page 3, line 57 an additional explanation is needed to see how the value of ~ 200 V/um was came from.”

We thank the referee for pointing out the fact and note that there was an error in terms of the reference cited which has been now changed (ref 16 and 17 have been swapped). The origin of the high electric fields lies in the in-built potential that forms as a result the heterojunction formed between the p an n type organic semiconductors, as experimentally verified in reference 16. Such high fields are expected at the junction of abrupt heterojunctions and this is further discussed within the manuscript (page 3 lines 60-63).

“The formation of nanoscale diodes throughout the volume of the device, in close proximity to the NPs leads to an in-built depletion region, with local electric fields as high as ~200 V μm^{-1},[16] which has been experimentally quantified with Fourier-Transform IR-absorption spectroscopy for the P3HT:PCBM system.”

R1 comment 4

“The authors need to explain why the sensitivity was improved compared to the reference below.A. Ciavatti et al. Dynamics of direct X-ray detection processes in high-Z Bi2O3 nanoparticles-loaded PFO polymer-based diodes. Appl. Phys. Lett. 111, 183301 (2017)”

We are thankful to the reviewer for highlighting this work. The origins for the better sensitivity observed in our work as opposed to that reported in the highlighted manuscript has now been included in our revised manuscript (lines 113-118) as follows:

“As opposed to the recently published Bi_2O_3 NP loaded PFO polymer based diodes,[28] the sensitivity obtained in this work is an order of magnitude higher due to the combination of the use of the BHJ thick films which enables a more balanced carrier transport, and the high NP loading, to increase the X-ray attenuation. The combination of the direct conversion of X-ray photons to charge carriers as well as the very high electric fields at the hybrid interfaces adjacent to the NPs, is a necessity for the much improved currents observed.”

R1 comment 5

“At page 7, lines 127-134 PC70BM is not suitable acceptor for fabricating a flexible detector. If

you consider the thermal and mechanical stability, it is better to choose from non-fullerene acceptors. Probably, the sensitivity of the proposed detector was decreased after undergoing 10 cycles of ~ 3 mm curvature. Please refer to the following references. T. Kim et al. Flexible, highly efficient all-polymer solar cells. Nat. Comm. 6, 8547 (2015)"

While we agree with the reviewer that $PC_{70}BM$ may not be an ideal acceptor, at this point, we are unable to confirm whether the observed decay is to due $PC_{70}BM$ alone or whether there would be other factors involved, such as degradation at the contacts. As a result, we have modified the discussion to cover all such possible aspects in the following manner (lines 151 –156):

"A slight variation in the rise and decay constant was observed possibly due to the poor mechanical properties of the contact materials or the use of a fullerene based acceptor, as opposed to an all polymer based BHJ, which might have deteriorated the mechanical properties of the BHJ[38]. Based on our concept of hybrid organic-inorganic materials for X-ray detectors, many new and more suitable combinations can now be examined for future detectors."

R1 comment 6

"In Figure 5 Bi2O3-40 showed relatively high carrier mobility. The authors need to explain the reason."

The high mobility observed for the Bi_2O_3-40 samples is primarily due to the higher crystallinity observed under that specific nanoparticle loading conditions. We apologise if this was not made clear in the manuscript. The following section has now been added to the discussion (lines 227 - 230) to emphasis this.

"The Bi_2O_3-40 device exhibits the highest charge carrier mobility, which is in agreement with the high crystallinity observed in the GI-WAXS as explained previously, as well as through DSC given in Supplementary Information 8 where a high crystallinity in excess of 45% is observed."

R1 comment 7

"Please provide a title that more accurately. It is better to add 'Bi2O3' and 'P3HT:PC70BM'."

We would like to note that although we have indicated the performance for a specific combination of organic semiconductors and high Z nanoparticle, we believe that the concept can be easily expanded to other combinations of materials that enable the same application. Therefore, in this instance while we do understand and respect the reviewer's suggestion, we do not think that it would be appropriate to include the materials into the title as suggested by the reviewer. As discussed above, the generic concepts proposed allows for a new type of hybrid X- ray detector to be designed and we would encourage all readers to target high sensitivity broadband detector designs based on the novel concept proposed.

Reviewer #2 (R2):

R2 comment 1

"There are many parameters to calculate the X-ray sensitivity. The parameter values for the calculated sensitivities in this work should be further justified"

We agree with the reviewer's comment that there is a number of ways to present sensitivity, as evident from the literature. Our preferred definition or method is based on the argument that the sensitivity depends on the amount of X-rays stopped, which depends on both the pixel area and its thickness, and hence, we have used the number of charges generated per incident dose per unit volume of the detector. We have now clearly defined this in the revised manuscript and thank the referee for requesting for clarity (page 5 lines 107-110)

"The X-ray sensitivity (S) depends on the amount of X-rays stopped, which depends on both the device cross section and its thickness, and hence, the sensitivity of the detector is calculated by: $S=(\int[I_{(X\text{-}ray)}(t) - I_{dark}]dt)/(D \times V)$...*"*

R2 comment 2

"In Fig 2(c), the measured sensitivity increases with increasing bismuth oxide NP loading. Does this mean that the sensitivity of the X-ray detector would increase with more loading of bismuth oxide NPs? In addition, is this behavior consistent with the theory?"

As the NP loading increases the dose deposited (or attenuated) in the detector volume is expected to increase (as shown theoretically in Figure S5), which is also in agreement with the detector sensitivity results (Figure 2 (c)). The low detector thickness means only a small percentage of the incident X-rays are attenuated, and attenuation in this case is primarily due to the bismuth oxide loading. In practical terms, the highest NP loaded device which could be fabricated using the given procedure is Bi_2O_3-80, due to the cracking of the film drying process at higher loadings. Therefore, at this stage we are unable to measure the sensitivity of devices beyond a Bi_2O_3-80 loading. We thank the referee for the comment and have added a few lines to fully discuss this point in the manuscript. See page 5 lines 88-92.

"The NP loading within the device active volume was increased in order to increase the X-ray attenuation by varying the Bi_2O_3 *content in the parent solution (0, 10, 20, 30, 40, 50, 60, 70 and 80 mg* ml^{-1}*: X mg* ml^{-1} *is noted as* Bi_2O_3*-X). It is worth to note that the highest NP loaded device which could be fabricated using the given procedure is* Bi_2O_3*-80, due to the formation of cracks during the annealing process at higher loadings beyond* Bi_2O_3*-80. However, with the appropriate selection of the organic bulk heterojunction and with tuning of the solvent used, much higher* Bi_2O_3 *loaded device fabrication maybe possible."*

R2 comment 3

"EDX imaging in Fig 4(b) looks to be uniform in a macroscopic large scale. How about the other samples containing more Bi2O3 NP loading? Real distribution of NPs may be non-uniform in a microscopic scale. Additional data to confirm the uniform distribution of Bi2O3 NPs are useful and necessary."

We agree with the reviewer that the EDX images listed in Figure 4 (b) are at the macroscopic scale.

To view the distribution on a microscopic scale, as requested by the reviewer, would require characterisation on a nanoscopic scale (such as using a Transmission Electron Microscope (TEM)). While the microscopic distribution will be important for future optimisation of charge transfer (for example to optimise colocation of the Bi_2O_3 with the BHJ interfaces), the main aim for this work was to confirm an even distribution of bismuth oxide throughout the volume of the device which will allow charge generation throughout the bulk of the device which can be then efficiently extracted using the organic BHJ. Our original intention in carrying out the EDX analysis was to indicate that no nanoparticle sedimentation or aggregation occur in our devices, as opposed to previous observations reported in the literature, ensuring that the BHJ system is utilised to its fullest extent. Consequently, we believe that the in-depth characterisation of the exact localisation of the nanoparticles with respect the BHJ interfaces, for the different loadings, will be a study of its own requiring extensive characterisation and optimisation. This would be outside of the scope of this study to prove that the devices work in principle, but will be considered for a fuller optimisation investigation in the future. We have modified the text to clarify this point, and will report in the future with a full structural study containing nanoscale information. See page 10 lines 205-209.

"Energy dispersive X-ray analysis carried out on the BHJ-NP film cross sections (Figure 4b) shows a uniform Bi_2O_3 NP distribution throughout the device thickness, indicating a homogeneous distribution of NPs and minimal dead volume without any NP sedimentation unlike previous reports.[10] Such distribution of the NPs offer an efficient X-ray to charge direct conversion, throughout the entirety of the device thickness."

R2 comment 4

"In general, one of main problems for organic devices is their bad stability. How about the X-ray sensors in this work? Have you checked the stability of the X-ray sensors used in this work?"

In terms of the stability under X-ray irradiation, we have so far not carried out extensive investigations as to what doses can be tolerated prior to any noticeable degradation of the semiconductor. Thus far, we have not observed instability in any of the detectors measured under experimental conditions specified for the material system. However, we have indicated the stability of the devices studied here based on a limited dose exposure (stated in lines 181- 184). A more in-depth study is required in order to fully understand the degradation processes and its impact on the device performance.

"It is noted that the Bi_2O_3-40 device did not show a noticeable performance degradation when exposed to 6 and 15 MV X-rays over several X-ray exposure cycles which results in a cumulative exposure dose of 0.15 Gy."

R2 comment 5

"Which sample is used for performance comparison shown in Fig 1(a)? is the same sample used to check good broad-band detection?"

We are thankful to the reviewer for pointing out the lack of clarity in this regard. For Fig 1(a), Bi_2O_3-40 and Bi_2O_3-80 devices were used for the 50 kV data points and Bi_2O_3-40 device was

used for 6 and 15 MV X-rays. This has been now indicated in the figure 1 (a) caption (page 4 line 76).

R2 comment 6

“The authors mention that the experimental and theoretical data in Fig 6(c) are consistent only with an order of magnitude difference. But, I see the difference is much bigger.”

We partially agree with the comment made by the reviewer, where the difference between the experimental and theoretical data is slightly higher (not “much bigger”) than an order of magnitude. Therefore, we have altered the statement in page 16 line 296-298.

“The GI-SAXS results are in good agreement with the predictions based on the Mie scattering effects, with approximately an order of magnitude difference in X- ray scattering at angles $<0.75^{\circ}$ for the d=20 and 40 nm NP dimensions.”

R2 comment 7

“Is there a relation between sensitivity and broad band detection in this sample?”

So far, we have not investigated as to whether there exists a relation between the sensitivity and the broadband detection. One assumption is that Mie scattering is dominant even under hard X-rays due to surface irregularities in the nanoparticles. However, in the absence of stronger experimental evidence at a more fundamental level to prove such a statement, we believe it to be best to avoid arriving at a definitive conclusion at this point. Experiments are on-going to clarify the relationships between the sensitivity and broad-band.

Minor comments

“1. The photos in Fig 2(f) are unclear.”

- The photos in Figure 2f have been replaced.

“2. Explain solid lines in Figures (for example, Fig 5 (b,d)).”

- An explanation has been added for the solid line in Figure 5(b) in page 13 line 242. The solid line in Figure 5(d) is already explained in page 13 line 244, as a fit to the Hecht equation.

Reviewers' comments:

Reviewer #1 (Remarks to the Author):

1. Compared to the previous works of you and other authors, it is better to present the title more clearly. Among the various methods of implementing the hybrid detector, this study showed the method about mixing Bi2O3 in the P3HT:PCBM organic active layer. It is appropriate to present the title containing 'Bi2O3' and 'P3HT:PCBM or other word'.

2. In Fig 1(a), the NP loading within the device was increased in order to increase the sensitivity of the proposed detector in soft X-ray region. In hard X-ray region, the sensitivity of the detector containing Bi2O3-40 was shown. Any results with Bi2O3-80? The NP loading within the active layer is still effective to increase the sensitivity?

3. In Fig 1(a), please explain why the similar sensitivity appeared despite the difference in the amount of NPs and the formation of BHJ in the active layer, compared the case of Bi2O3-40 with the results presented in ref[28].

Reviewer #2 (Remarks to the Author):

The authors have addressed all my concern and improved a lot comparing with last version.

Therefore, I think this work can appear on NC.

Submission of revised manuscript (NCOMMS-18-07706-A): ***"High sensitivity organic-inorganic hybrid X-ray detectors with direct transduction and broadband response".***

We are grateful to your comments, which we believe have improved the context, clarity and quality of the manuscript.

The points raised by the Reviewer 1 are addressed in full below. These changes have also been highlighted in yellow in the revised manuscript submitted with this letter.

Reviewer #1 (R1):

In the previous revision, Reviewer #1 raised 7 comments in total, and we addressed all of these points with modifications for Comments 1-6. The previous comment 7 is based on the alteration of the title and we already responded accordingly.

R1 comment 1

"Compared to the previous works of you and other authors, it is better to present the title more clearly. Among the various methods of implementing the hybrid detector, this study showed the method about mixing Bi2O3 in the P3HT:PCBM organic active layer. It is appropriate to present the title containing 'Bi2O3' and 'P3HT:PCBM or other word."

We thank the referee, but note this comment was addressed in the previous reply. It should be noted that although we have indicated the performance for a specific combination of organic semiconductors and high Z nanoparticle, we believe that the concept can be expanded to other combinations of materials that enable the same application. Therefore, in this instance while we do understand and respect the reviewer's suggestion, we do not think that it would be appropriate to include the materials into the title as suggested by the reviewer. As discussed above, the generic concepts proposed allows for a new type of hybrid X-ray detector to be designed and we would encourage all readers to target high sensitivity broadband detector designs based on the novel concept proposed. It is also the expected style for Nature journal papers.

R1 comment 2

"The authors should describe in the manuscript why the authors selected the P3HT and "In Fig 1(a), the NP loading within the device was increased in order to increase the sensitivity of the proposed detector in soft X-ray region. In hard X-ray region, the sensitivity of the detector containing Bi2O3-40 was shown. Any results with Bi2O3-80? The NP loading within the active layer is still effective to increase the sensitivity?"

In our experiments, we have discussed the sensitivities as well as the rise and decay times for 9 different Bi2O3 loadings. While the Bi2O3-80 device demonstrated the highest sensitivity under soft X-rays, these detectors also exhibited a very slow rise and decay time under soft X-ray irradiation. On the other hand, the Bi2O3-40 detector showed faster time constants with an excellent high sensitivity. Therefore the Bi2O3-40 detector was selected for the measurements under hard X-rays that were conducted in the Royal Surrey County Hospital under 6 and 15 MV LINAC. No Bi2O3 -80 samples were tested.

R1 comment 3

"In Fig 1(a), please explain why the similar sensitivity appeared despite the difference in the amount of NPs and the formation of BHJ in the active layer, compared the case of Bi2O3-40 with the results presented in ref[28]."

Although similar sensitivity appeared between our work and ref 28, there are several points which make the analysis different.

In ref 28, the PFO-Bi_2O_3 based detector was tested at 35 kV Mo target resulting in a detector sensitivity of 160 $\mu CmGy^{-1}cm^{-3}$. In comparison our detectors were measured under 50 kV tungsten X-ray source, with a sensitivity of 105 $\mu CmGy^{-1}cm^{-3}$. As stated and shown schematically the X-ray attenuation significantly improves by approximately one order of magnitude as the X-ray spectrum shifts from 50 kV to 35 kV. Therefore, measurement of our detectors under a 35 kV Mo target would result in at least an order of magnitude improvement (~1000 $\mu CmGy^{-1}cm^{-3}$) in sensitivity which is better than that measured in ref 28.

We have now added this discussion to the manuscript in page 6 lines 113-124.

"We note that Ciavatti et al.[28] recently reported Bi_2O_3 NP loaded PFO polymer based diodes for X-ray detection where the highest S observed was 160 $\mu C\ mGy^{-1}\ cm^{-3}$ which is slightly higher than the S of Bi_2O_3-40 (105 $\mu C\ mGy^{-1}\ cm^{-3}$). Despite the similarity in magnitude of these S values, we note that the former work employed a 35 kV Mo target as opposed to the 50 kV W target used in this work. As stated and shown schematically (Figure 1a), the X-ray attenuation significantly improves by approximately one order of magnitude as the X-ray spectrum shifts to lower energies which is expected to result in the nearly similar S values for the Bi_2O_3 NP loaded PFO polymer based diodes and the Bi_2O_3-40 diodes. We expect the S values for the Bi_2O_3-40 diodes to be higher if measured under low kV sources due to the enhanced X-ray attenuation as a result of increase in the mass attenuation, especially under high NP loading as well as due to the use of the BHJ thick films which enables a more balanced carrier transport."
